# “Dictionary of immune responses” reveals the critical role of monocytes and the core target IRF7 in intervertebral disc degeneration

**DOI:** 10.3389/fimmu.2024.1465126

**Published:** 2024-10-17

**Authors:** Peichuan Xu, Kaihui Li, Jinghong Yuan, Jiangminghao Zhao, Huajun Pan, Chongzhi Pan, Wei Xiong, Jianye Tan, Tao Li, Guanfeng Huang, Xiaolong Chen, Xinxin Miao, Dingwen He, Xigao Cheng

**Affiliations:** ^1^ Department of Orthopaedics, The Second Affiliated Hospital, Jiangxi Medical College, Nanchang University, Nanchang, China; ^2^ Jiangxi Provincial Key Laboratory of Spine and Spinal Cord Disease, Nanchang University, Nanchang, China; ^3^ Department of Stomatology, The Second Affiliated Hospital, Jiangxi Medical College, Nanchang University, Nanchang, China

**Keywords:** intervertebral disc degeneration, cytokine, monocytes, machine learning, single-cell sequencing, IRF7

## Abstract

**Background:**

Intervertebral disc degeneration (IDD) is widely regarded as the primary contributor to low back pain(LBP). As an immune-privileged organ, upon the onset of IDD, various components of the nucleus pulposus (NP) are exposed to the host’s immune system, accumulating cytokines. Cytokines facilitate intercellular communication within the immune system, induce immune cells polarisation, and exacerbate oxidative stress in IDD.

**Methods:**

Machine learning was used to identify crucial immune cells. Subsequently, Immune Response Enrichment Analysis (IREA) was conducted on the key immune cells to determine their cytokine responses and polarisation states in IDD. “CellChat” package facilitated the analysis of cell-cell communication. Differential gene expression analysis, PPI network, GO and KEGG pathway enrichment analysis, GSVA, co-expressed gene analysis and key gene-related networks were also performed to explore hub genes and their associated functions. Lastly, the differential expression and functions of key genes were validated through *in vitro* and *in vivo* experiments.

**Results:**

Through multiple machine learning methods, monocytes were identified as the crucial immune cells in IDD, exhibiting significant differentiation capacity. IREA revealed that monocytes in IDD polarize into an IFN-a1 and IFN-b enriched Mono-a state, potentially intensifying inflammation. Cell–cell communication analysis uncovered alteration in ANNEXIN pathway and a reduction in CXCL signaling between macrophages and monocytes, suggesting immune response dysregulation. Furthermore, ten algorithms identified three hub genes. Both experiments conducted *in vitro* and *in vivo* have conclusively shown that IRF7 serves as a crucial target for the treatment of IDD, and its knockdown alleviates IDD. Eight small-molecule drugs were predicted to have therapeutic potential for IDD.

**Conclusion:**

These findings offer a multidimensional understanding of the pathogenesis of IDD, pinpointing monocytes and key genes as potential diagnostic and therapeutic targets. They provide novel insights into potential diagnostic and therapeutic targets for IDD.

## 1 Introduction

Intervertebral disc degeneration (IDD), a prevalent degenerative condition, predominantly impacts middle-aged and elderly individuals, often resulting in chronic low back pain (LBP) (1). LBP affects an estimated 80% of the world’s population, significantly diminishing their quality of life and imposing a substantial economic toll on society (2). The intervertebral disc, situated between two adjacent vertebrae, comprises the annulus fibrosus (AF), nucleus pulposus (NP), and cartilaginous endplate (CEP) located above and below the vertebrae (3, 4). The NP, a gelatinous core, primarily comprises water, proteoglycans, and collagen. It withstands and distributes pressure while maintaining the height and hydration of the intervertebral disc through its high osmotic pressure properties (5).

During disc degeneration, the NP’s ability to synthesize proteoglycans and collagen decreases, leading to reduced moisture content and impaired pressure resistance. At the same time, there is an increased expression of inflammatory mediators and proteases within the NP, which degrade the extracellular matrix (ECM), further compromising the structural integrity of the disc (6, 7). These biochemical changes can also trigger or exacerbate the inflammatory response, creating a vicious cycle that accelerates disc degeneration (8). Thus, it is essential to explore the pathological mechanisms of IDD and identify targets for nucleus pulposus cells (NPCs) dysfunction to develop effective diagnostic and therapeutic strategies for IDD.

Since its formation, the NP has been encapsulated by the AF and the CEP, creating a unique structure that isolates the NP from the host’s immune system. Thus, the intervertebral disc is recognized as an immune-privileged organ (9). During IDD, various components of the NP can elicit an autoimmune response upon exposure to the host’s immune system, resulting in vascular infiltration and the aggregation of inflammatory cytokines that disrupt the homeostasis of the disc (10, 11).

Cytokines, a wide range of small secreted proteins, bind to homologous receptors on target cells, mediate intercellular communication within the immune system, and are essential therapeutic targets (12, 13). Natural disc cells produce numerous cytokines up-regulated during IDD, driving many metabolic processes of disc degeneration (14–16). Phillips et al. have shown that NPCs produce multiple cytokines and chemokine receptors and can exhibit paracrine and autocrine response modes (17). These cytokines further promote IDD progression by activating intracellular signaling pathways (18, 19). During IDD progression, these cytokines may up-regulate neurotrophic and angiogenic factors, leading to angiogenesis and innervation (20, 21). Thus, the role of cytokines in the progression of IDD cannot be ignored. Cytokine-based therapies and antagonists treat various diseases, including cancer and autoimmune diseases (22). Numerous studies have highlighted the central role of cytokines in immunity. However, previous studies have often lacked a comprehensive view of each immune cell type for each cytokine. Ang Cui et al. mapped the global view of “immune-cytokine” correspondences on a single-cell scale, creating a “Dictionary of immune responses” (22). This “dictionary” can identify the most active cytokines in disease and how different immune cells perform different functions depending on the cytokine signals they receive. Its emergence provides a new perspective for studying cytokine-immune cell polarisation in diseases and helps better understand the roles of immune cells in disease development.

In this study, a multifaceted bioinformatics approach was employed to explore the role of cytokine-driven monocytes polarisation in the immune system and identify essential genes that could impact the disease, suggesting potential therapeutic targets for alleviating IDD.

## 2 Methods

### 2.1 Transcriptome data sources

**Figure 1:**
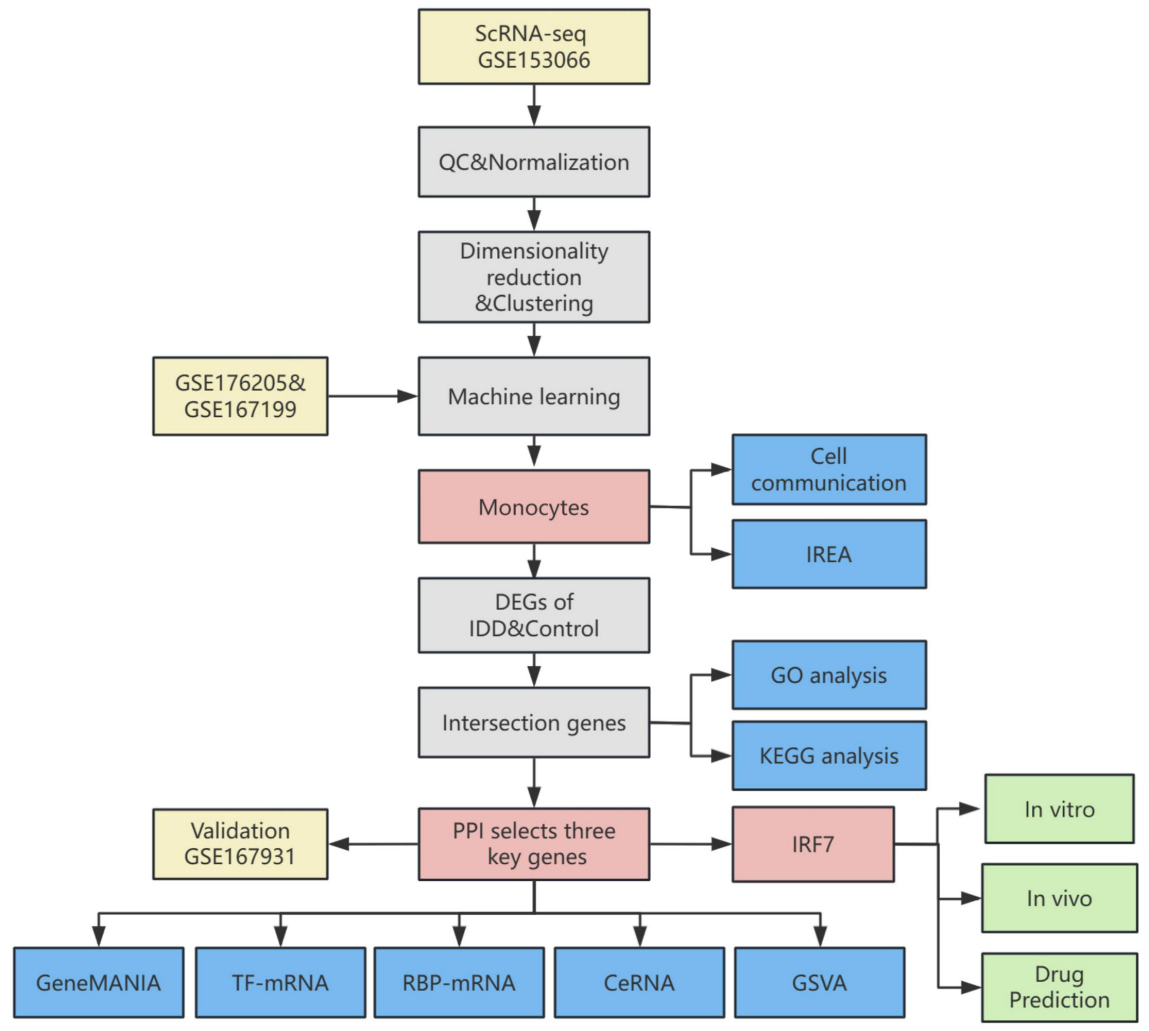
Flowchart of this study.

The flowchart depicted in **Figure 1** outlines the systematic approach undertaken in this study. All datasets employed for this research are accessible to the public, ensuring transparency and reproducibility. The transcriptome data were sourced from the publicly accessible Gene Expression Omnibus (GEO) database at https://www.ncbi.nlm.nih.gov/geo/. High-throughput sequencing data from GSE176205 and GSE167199 were corrected for batch effects using the “sva” package(version 3.42.0) in R (23). The sequencing platform for GSE176205 was GPL20301 Illumina HiSeq 4000 (Homo sapiens), which included 6 IDD and 3 standard control NP samples. The sequencing platform for GSE167199 was GPL24676 Illumina NovaSeq 6000 (Homo sapiens), including 3 IDD and 3 standard control NP samples. GSE167931 was utilized as an additional dataset for validation purposes, sequenced on the GPL20795 HiSeq X Ten (Homo sapiens) platform, and included 5 IDD and 4 standard control NP samples.

### 2.2 Download and processing of single-cell sequencing data

A single-cell sequencing dataset was retrieved from the GEO database, which contained 16 samples (8 IDD and 8 standard control NP samples). The “Seurat” R package(version 4.2.0) was employed in the R to import the raw data within GSE153066 from the database (24). Filtering out low-quality cells and genes involved applying stringent criteria to ensure data quality: 1) Cells that failed to express at least 200 genes were excluded. 2) Cells with a mitochondrial gene expression percentage below 25% were also retained. 3) Cells with fewer than 4,000 expressed genes were retained. 4) Cells with a UMI reading of fewer than 10,000 were retained. The data were normalized using the “Seurat” R package. Following normalization, balancing average expression and dispersion was employed to pinpoint highly variable genes. Principal component analysis (PCA) yielded significant PCs, which served as input for graph-based clustering. A harmony approach was used to eliminate batch effects across samples. For clustering, the “FindClusters” function, leveraging the optimized Shared Nearest Neighbor (SNN) modular clustering algorithm, was applied to identify 16 distinct clusters with a resolution of 0.5. The “RunUMAP” function was used for Uniform Manifold Approximation and Projection (UMAP). UMAP-1 and UMAP-2 visualized cell aggregation, subsequently enabling the identification of cell clusters based on their type-specific biomarkers. The “RunTSNE” function was then used for t-distributed stochastic neighbour embedding(t-SNE). t-SNE-1 and t-SNE-2 visualized T cell aggregation.

### 2.3 Single sample gene set enrichment analysis (ssGSEA)

ssGSEA represents an extension of Gene Set Enrichment Analysis(GSEA) and it was employed to calculate enrichment scores for each cell type in conventional transcriptome data based on a list of marker genes (25). Based on a list of marker genes for each cell type, we used “GSVA” R package (version 1.42.0) to perform ssGSEA analysis to calculate the enrichment fraction of each cell type in transcriptome data.

### 2.4 Machine learning

Support Vector Machine-Recursive Feature Elimination (SVM-RFE), a machine learning approach, trains subsets of features across categories to refine the set and pinpoint the most predictive genes. Utilizing the “glmnet” package in R (version 4.1.4), LASSO regression was implemented to compute and select linear models, effectively retaining only informative variables. Random forest analysis was conducted using the “RandomForest” function, where the minimum error was chosen as the mtry node value, and the stabilized image value was selected as the ntree. The results of SVM-RFE, LASSO regression, and Random forest were combined to select the key immune cells in this study.

### 2.5 Immune response enrichment analysis

Immune Response Enrichment Analysis (IREA) is a method Ang Cui et al. proposed to infer cytokine activity and immune cell polarisation during immunization (26). In a cell type-centric view, IREA uncovers over 66 distinct cytokine-mediated polarisation states within immune cell types, encompassing novel and previously undescribed states. IREA was used to calculate the cytokines that primarily generate a response based on the differentially expressed genes (DEGs) of vital immune cells and the polarisation states of critical immune cells in IDD.

### 2.6 Cell-cell communication analysis and ligand-receptor expression

Using the “CellChat” (version 1.1.3) R package, CellChat objects were constructed (27). Cell-cell communication was analyzed using the “CellChatDB.human” ligand-receptor interaction database as the reference. The “mergeCellChat” function was employed to combine the CellChat objects of each group, enabling a comparison of the interaction number and the interaction strength. The “netVisual_diffInteraction” function was used to visualize differences in the number or strength of interactions between groups and different cell types. Finally, the “netVisual_aggregate” function was used for visualization.

### 2.7 Identification of differentially expressed genes

The DEGs were identified utilizing the “limma” R package (version 3.50.0) (28). The screening criteria were |log2Fold Change|>1 and adjusted p-value <0.05. Heatmaps were created employing the “pheatmap” R package(version 1.0.12) and clustered using euclidean distance and hierarchical clustering methods. Further, we explored the differences between the two groups in single-cell data. The “FindAllMarkers” function in the “Seurat” R package was employed to identify DEGs of vital immune cells between two groups.

### 2.8 Protein-protein interaction network construction

A PPI network was constructed by utilizing the Search Tool for the Retrieval of Interacting Genes (STRING) online resource (29). Sub-networks were constructed for proteins with interaction scores greater than 700, from which proteins with direct interactions with intersecting DEGs were extracted and analyzed. Afterward, PPI network analysis was executed using the Cytohubba plug-in (30). Ten algorithms (Betweenness, BottleNeck, Closeness, Degree, EcCentricity, EPC, MCC, MNC, Radiality and Stress) were used to screen the top 100 proteins in terms of importance. The intersecting proteins from these algorithms and the genes identified in the intersecting DEGs were designated as key genes.

### 2.9 Gene ontology (GO) and kyoto encyclopedia of genes and genomes(KEGG) pathway enrichment analysis

GO enrichment analysis includes three domains: biological process (BP), molecular function (MF), and cellular component (CC) analysis (31). The KEGG is a bioinformatics resource for identifying enriched and significantly altered metabolic pathways within gene lists (32). The genes of interest in IDD were analyzed for GO and KEGG enrichment using the R package “clusterProfiler” (version 4.2.2) (33).

### 2.10 GeneMANIA

The GeneMANIA website (http://genemania.org) forecasts associations between functionally analogous genes and pivotal genes (34). Gene interaction networks for key genes were constructed using the GeneMANIA website.

### 2.11 Gene set variation analysis (GSVA)

To explore the disparities in biological functionalities between the two groups, the gene set “h.all.v2023.1.Hs.symbols.gmt” sourced from the MSigDB was employed as the reference gene set. GSVA was performed using the R package “GSVA” (version 1.42.0).

### 2.12 Construction of ceRNA network, RBP-mRNA network, mRNA-TF network

Since the mechanism of action of competing endogenous RNA (ceRNA) in IDD remains unclear, we used miRTarBase (https://mirtarbase.cuhk.edu.cn/~miRTarBase/miRTarBase_2022/php/index.php), starbase3.0 (https://rnasysu.com/encori) and miRDB databases (https://mirdb.org/index.html) to back-predict microRNAs of key genes and to predict lncRNAs of common microRNAs of key genes, constructing a ceRNA network (35, 36).

The open-source platform (https://starbase.sysu.edu.cn/tutorialAPI.php#RBPTarget) was used to investigate mRNA and RNA-binding protein (RBP) expression associations.

The mRNA-TF interaction relationships between key genes were constructed using the TRRUST(Transcriptional Regulatory Relationships Unraveled by Sentence-based Text Minmining) database. Networks were constructed using Cytoscape(version 3.9.1).

### 2.13 Cell culture and processing

We acquired human NPCs from Procell Life Science & Technology Co., Ltd (CP-H097, Wuhan, China). We cultured in F12/DMEM medium (Gibco, USA) supplemented with 1% Penicillin-Streptomycin Solution (NCM Biotech, China) and 10% fetal bovine serum (FBS; Gibco, USA). As described in previous literature, NPCs were induced using 100 µM TBHP (Sigma-Aldrich, St. Louis, MO, USA) for 4 hours to construct the IDD model *in vitro* (37). Cells were transfected with small interfering RNA (si-RNA) to knock down IRF7 (ENSG00000185507, Gene ID: 3665) using the riboFECT CP Transfection Kit (RiboBio, Cat. No C10511-05, China).

### 2.14 RNA extraction and PCR

Total RNA was extracted from the cells using TRIzol (Thermo Fisher, USA) and chloroform (Thermo Fisher, USA). The purity and concentration of RNA samples were quantitatively assessed utilizing a Nanodrop One spectrophotometer (Thermo Fisher, USA). cDNA synthesis from the extracted RNA was performed using the PrimeScript RT reagent. Reverse transcription reagents were purchased from TaKaRa, and an RT-qPCR assay was conducted using an ABI 7500 Real-Time PCR machine (Thermo Fisher, USA). **Supplementary Table 1** provides the primer sequences employed in the study.

### 2.15 Animal model

The Ethics Committee of Nanchang University approved all experimental procedures involving animals, and all procedures followed the ARRIVE guidelines. Male Sprague-Dawley (SD) rats, aged 12 weeks, were sourced from SpePharm Biotechnology Ltd. (Beijing, China). A rat tail puncture model was established according to the protocol described in the literature (38). Rats were administered 4% sodium pentobarbital (1mL/1kg) to induce anaesthesia. Following skin sterilization, a 21-gauge needle was employed to make a vertical puncture into the Co4/5 disc of the tail, reaching a depth of approximately 5 millimeters. Upon completing the puncture, the needle was rotated in a full 360-degree motion and maintained in position for one minute. On postoperative days 1, 7, and 14, si-RNA and control agent si-NC 5 nmol (10 µL) were delivered directly into the disc along the original puncture channel. The health status of the rats was monitored daily after surgery. The flowchart of the animal experiment was performed by Figdraw (www.figdraw.com).

### 2.16 X-ray and magnetic resonance imaging (MRI)

X-rays and MRIs of the caudal intervertebral discs of rats were performed at 4 weeks postoperatively. The disc height index (DHI) score was utilized to quantify the structural integrity of the discs. This score was derived from the ratio of the intervertebral space height to the height of the neighbouring vertebral bodies observed in X-ray images. Additionally, the severity of IDD was assessed using MRI images and analyzed with the Pfirrmann grading system as a standardized reference.

### 2.17 HE and SO&FG staining

4 weeks post-surgery, the rats were euthanized, and their disc samples were fixed and immersed in 4% paraformaldehyde. Following the EDTA decalcification solution, the disc samples were dehydrated, embedded in paraffin, and sectioned serially. NP morphology was examined using hematoxylin-eosin (HE) and Safranin O-Fast Green (SO&FG) staining.

### 2.18 Immunohistochemical staining

Sections were treated with a 3% hydrogen peroxide solution to block endogenous peroxidase activity, followed by 3% bovine serum albumin (BSA; Servicebio, Cat no: GC305010). Primary antibodies used were COL2A1(Proteintech,Cat No.28459-1-AP), MMP13(Proteintech,Cat No.18165-1-AP), NLRP3(Proteintech,Cat No. 19771-1-AP) and IL-1β(Proteintech,Cat No. 16806-1-AP). These were incubated overnight at a temperature of 4°C. HRP-coupled secondary antibodies (Servicebio, Cat no: GB23303) were then used for the corresponding primary antibodies. Finally, the samples were stained using diaminobenzidine and hematoxylin to label the nucleus.

### 2.19 Immunofluorescence

The NPCs were fixed with 4% Paraformaldehyde Fix Solution (Biosharp, China, Cat no: BL539A) for 15 minutes. The cells were blocked with 3% BSA at room temperature for 30 minutes. Then, the cells were incubated overnight with antibodies against COL2A1, MMP13,NLRP3 and IL-1β. After washing the samples, the secondary antibody was added and incubated at room temperature for 50 minutes (Servicebio, Cat no:GB21303). The samples were rewashed, and DAPI staining solution was added for incubation at room temperature in the dark for 10 minutes.

### 2.20 Small molecule drug prediction and molecular docking

Based on the top 20 up-regulated and 20 down-regulated genes with significant differences between the IDD group and the control group, small molecule compounds that might be effective in treating IDD were predicted using the Connectivity Map (CMAP) database. Eight compounds with the highest scores as potential drugs were selected, with toxic compounds excluded. We downloaded SDF format files for potential drugs from PubChem, PDB files of target proteins in the PDB database, and dehydrated and deliganded the proteins in PyMOL. Proteins were hydrogenated in AutoDockTools (version 1.5.7). Molecular docking was then performed in AutoDockTools. The strength of the binding energy serves as an indicator of the probability of the receptor and ligand binding together, with a decrease in binding energy corresponding to an increase in the affinity. The lower the binding energy, the more stable the conformation of the receptor became. The results of molecular docking were visualized in PyMOL.

### 2.21 Statistical analysis

Statistical analyses were performed utilizing R software (version 4.1.2). Spearman’s correlation test were employed to examine associations between two variables. The Wilcoxon rank-sum test was applied for comparisons between two groups, while the Kruskal-Wallis test was utilized for comparisons involving three or more groups. A statistical significance threshold was set at a p-value of less than 0.05.

## 3 Results

### 3.1 Single-cell dimensionality reduction clustering and annotation

**Figure 2:**
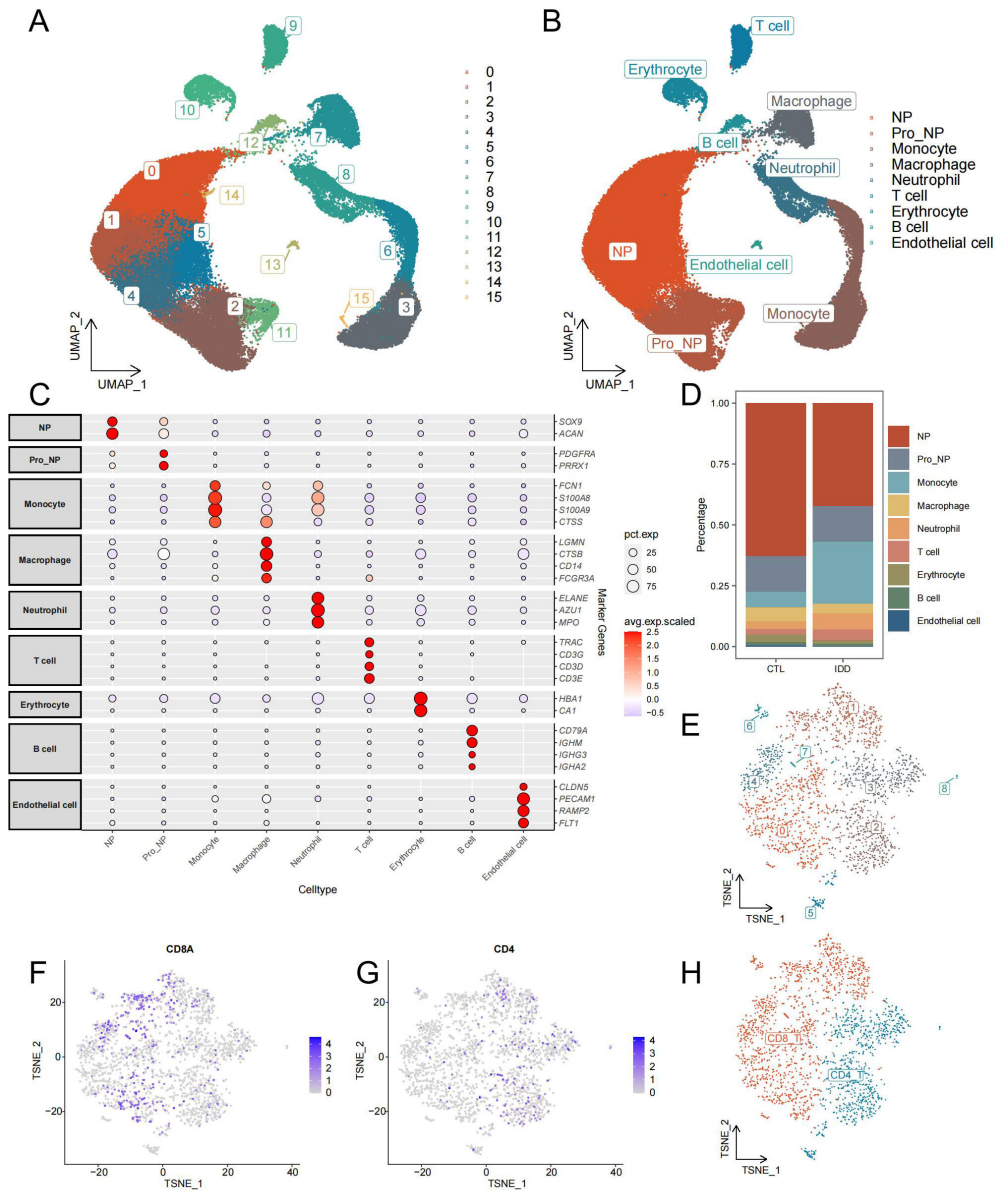
Identification of cell types from single-cell sequencing data. **(A)** The UMAP plot illustrates the clustering of all cells into 16 distinct clusters. **(B)** Another UMAP plot shows the annotation of these 16 clusters into 9 different cell types. **(C)** The annotation reference plot displays the expression of marker genes for each cell type. **(D)** A bar chart presents the proportion of different cell types in the IDD and control groups. **(E)** t-SNE plot showing the re-clustering results and distribution of T cells. **(F)** Distribution of CD8 in T cells. **(G)** Distribution of CD4 in T cells. **(H)** t-SNE plot demonstrating the annotation results of T cell subsets.

After initial quality control and doublet removal of IDD and control samples, 65,165 cells were obtained from the single-cell dataset. All cells were aggregated into 16 clusters (**Figure 2A**). And annotated by cell-specific biomarkers, we finally found 9 cell types, namely: nucleus pulposus (NP, cluster 0,1,4,5,14), progenitor nucleus pulposus (Pro_NP, cluster 2, 11), Monocyte (cluster 3,6,15), Macrophage (cluster 7), Neutrophil (cluster 8), T cell (cluster 9), Erythrocyte (cluster 10), B cell (cluster 12), and Endothelial cell (cluster 13) (**Figures 2B, C**). The distribution and number of different cell types varied significantly between the two groups (**Figure 2D**). NPCs were most reduced considerably in the IDD group, while Pro_NP, Macrophage, Erythrocyte, B cell, and Endothelial cell were also reduced to varying degrees. Monocytes increased most significantly in the IDD group, and Neutrophil also increased in IDD. T cells were re-clustered and categorized into CD4^+^ T cells and CD8^+^ T cells based on their distinct expression patterns (**Figures 2E–H**).

### 3.2 Screening of vital immune cells

**Figure 3:**
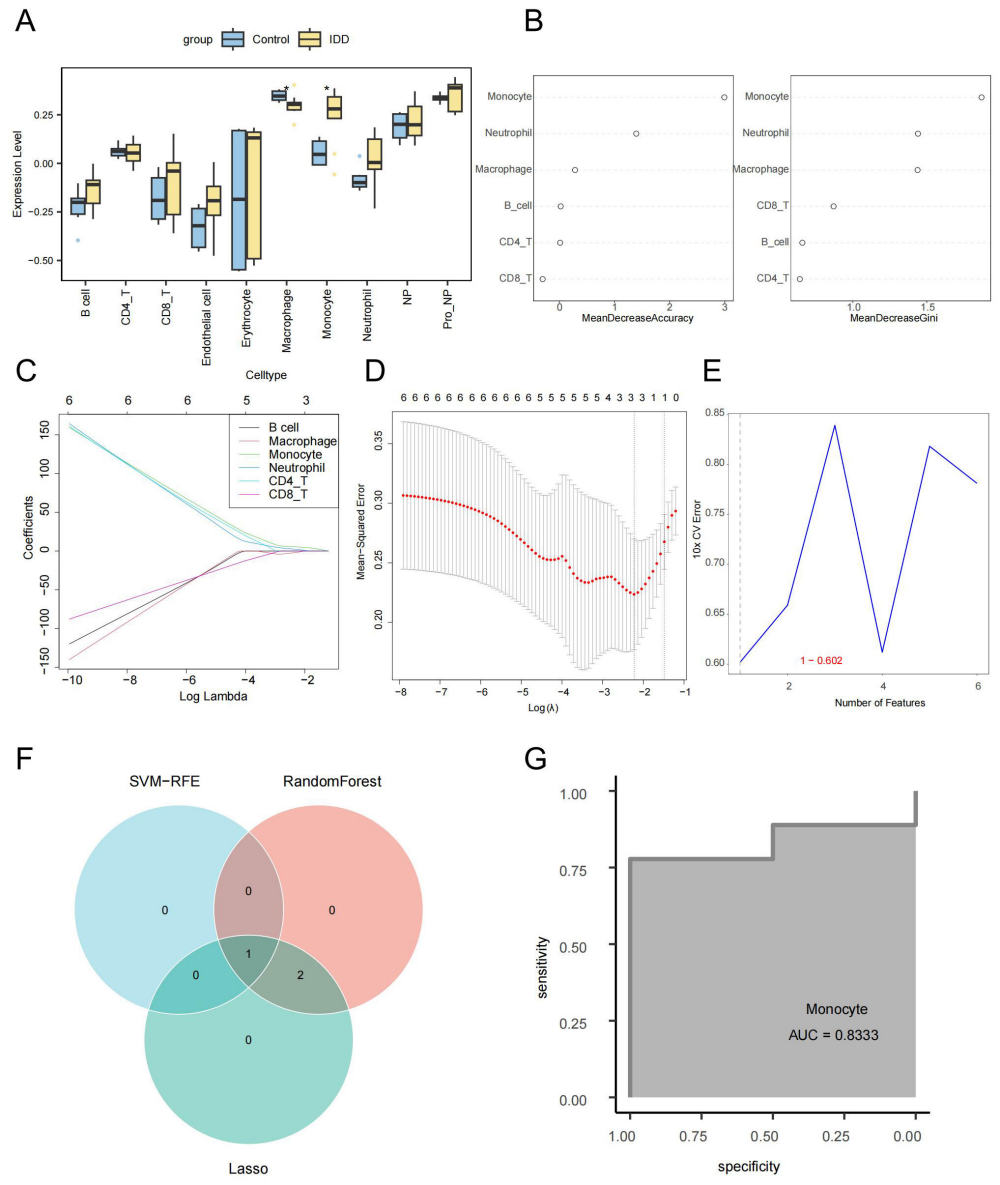
Screening of key immune cells via machine learning. **(A)** Box plot showing differences in cell enrichment scores between IDD and standard control samples. **(B)** The random forest analysis presents the ranking of the importance of six immune cell types in IDD from highest to lowest, Left: MDA; Right: MDG. **(C)** The coefficient distribution curve of each immune cell in the LASSO regression model. **(D)** Mean Squared Error in the LASSO regression model. **(E)** Error rate distribution in the SVM-RFE model. **(F)** The Venn diagram illustrates the identification of monocytes as key immune cells by three different machine learning algorithms. **(G)** The ROC curve confirms the critical role of monocytes in IDD.

The ssGSEA algorithm was used to calculate the enrichment scores of each cell type (**Supplementary Table 2**). There was a notable decrease in the number of macrophages in the IDD group compared to the control group. In contrast, the IDD group’s monocytes count was significantly elevated compared to the control group. This is consistent with trends observed in the single-cell data (**Figure 3A**). The Random Forest, LASSO regression, and SVM-RFE were used to screen for critical immune cells. We selected the top 3 immune cells using the random forest algorithm based on Mean decrease accuracy (MDA ) and Mean decrease Gini (MDG), which were macrophages, monocytes, and neutrophils (**Figure 3B**). By LASSO regression analysis, we obtained the same three types of immune cells (**Figures 3C, D**). However, through the SVM-RFE method, we only screened for monocytes (**Figure 3E**). Finally, the immune cells detected by each method were intersected, concluding that the most critical immune cell in IDD was the monocytes (**Figure 3F**). The ROC curve showed that the monocytes enrichment score had good efficacy for distinguishing IDD samples from normal control samples (AUC=0.8333, **Figure 3G**).

### 3.3 Immune response enrichment analysis of key immune cells

**Figure 4:**
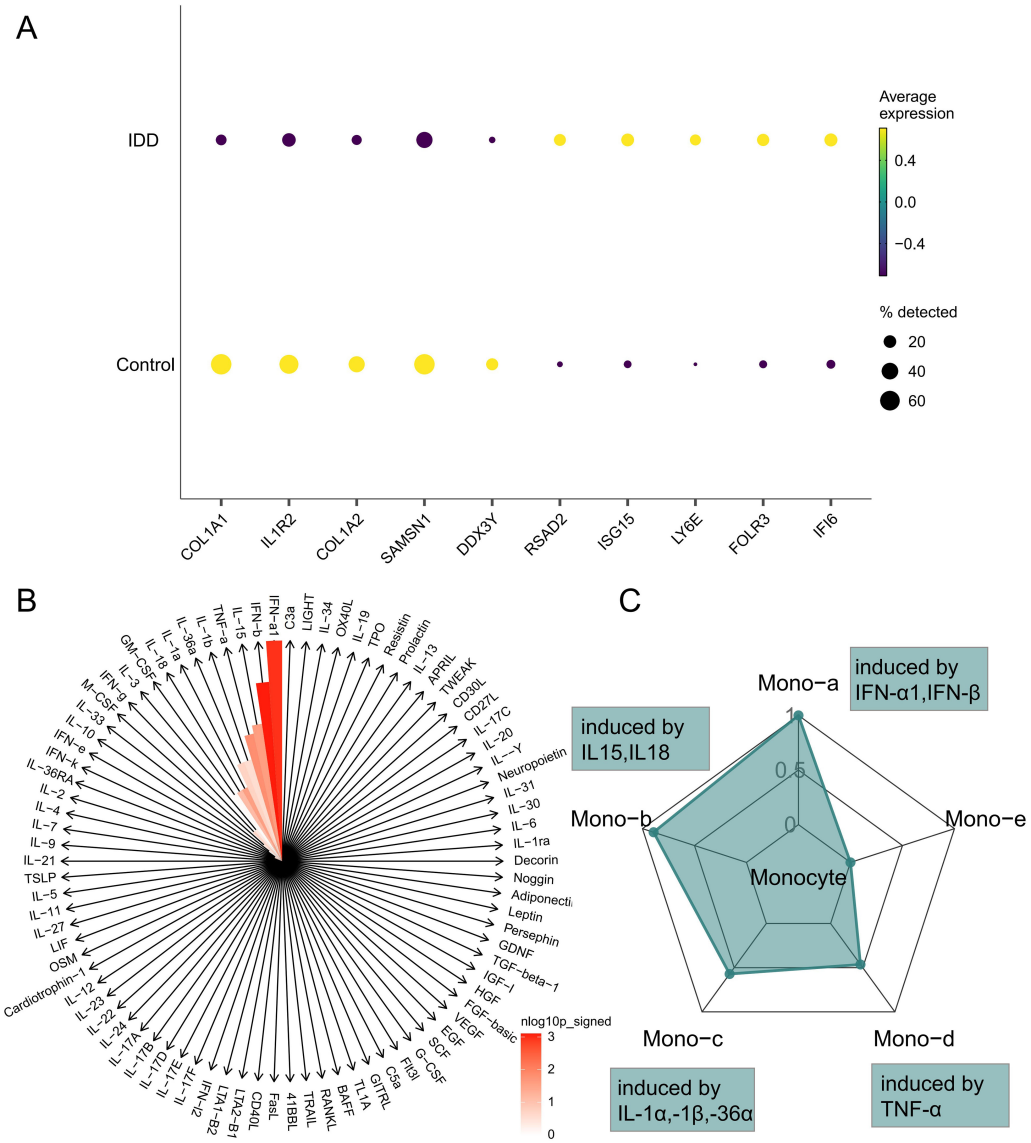
Immune Response Enrichment Analysis. **(A)** The bubble plot shows the top five genes with the most significant differences in Monocytes between IDD and normal controls. **(B)** The cytokine enrichment plot displays the cytokines significantly enriched in Monocytes in IDD. **(C)** The radar chart illustrates the polarization states of Monocytes in IDD.

In order to clarify the changes of cytokines in monocytes during IDD and to explore the polarization state of monocytes after receiving different cytokines, we performed a comparative analysis of monocytes between different groups to explore the immune status of monocytes in IDD (**Figure 4A**; **Supplementary Table 3**). Subsequently, IREA was conducted based on significantly up-regulated differentially expressed genes in IDD. The cytokine enrichment plot showed that monocytes in IDD were predominantly enriched for cytokines such as IFN-a1 and IFN-b (**Figure 4B**; **Supplementary Table 4**). The cellular polarisation radar plot indicated that monocytes were primarily in the Mono-a polarisation state (type I interferon-induced polarisation), which may exacerbate the IDD’s inflammatory response (**Figure 4C**; **Supplementary Table 5**). When IDD occurs, IFN-a1 and IFN-b are significantly enriched in the intervertebral disc. And the monocytes are in a Mono-a polarized state, which may lead to the aggregation of inflammatory factors and matrix degradation in the intervertebral disc.

### 3.4 Cell-cell communication analysis

**Figure 5:**
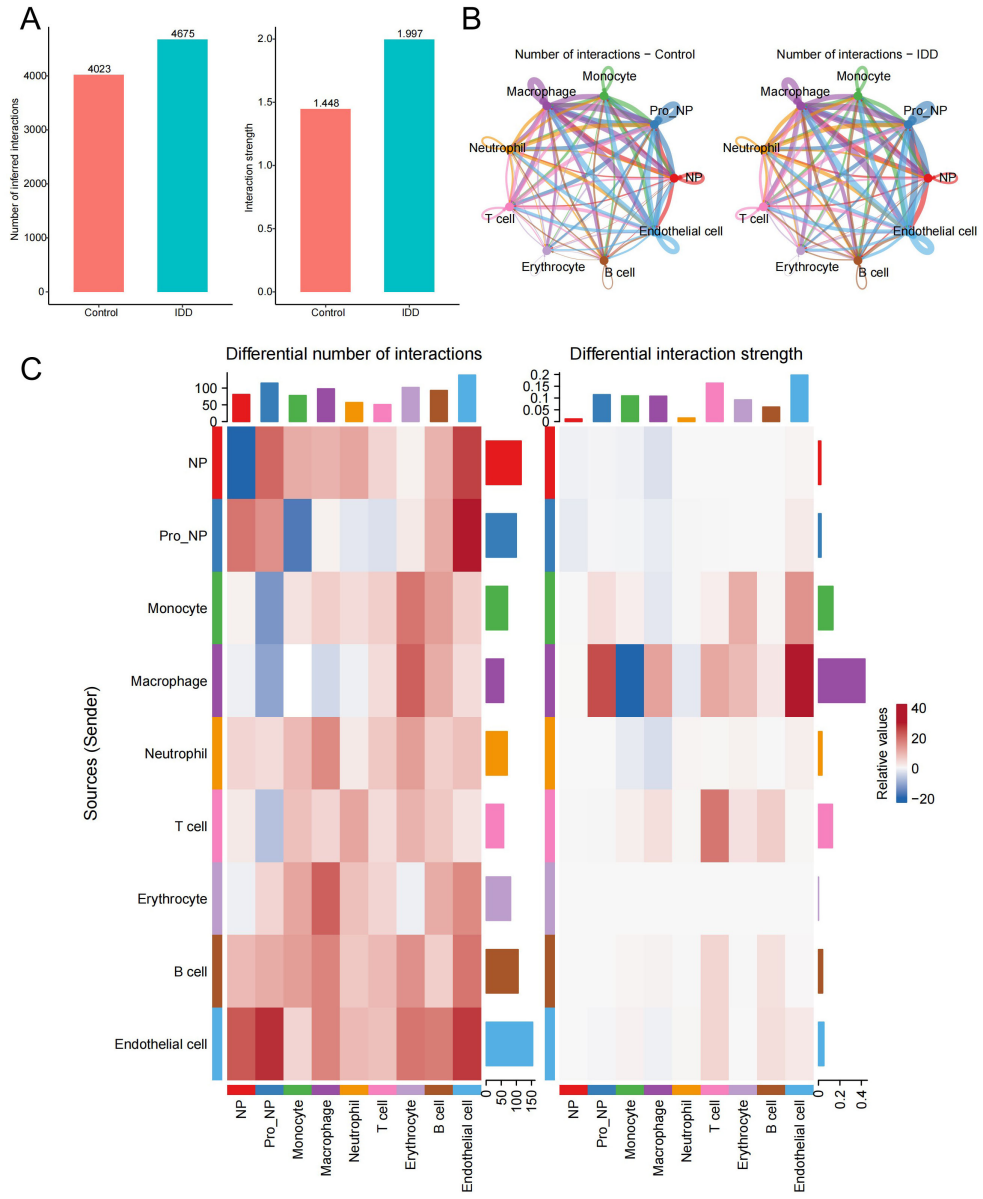
Cell-cell communication analysis. **(A)** The bar chart illustrates the differences in the total number and strength of intercellular communication signals between the control and IDD groups, with the left panel showing communication quantity and the right panel showing communication strength. **(B)** The network diagram displays the interaction counts between cell types in the control and IDD groups, with lines representing interactions between cells, colored according to cell types. Thicker lines indicate more interactions between groups, with the left panel for the control group and the right panel for IDD. **(C)** The heatmap shows changes in the number and strength of intercellular communication signals between cell types in the control and IDD groups. Red indicates an increase or enhancement in communication quantity/strength in the IDD group, while blue indicates a decrease or reduction. The left panel represents the number of communication and the right panel represents communication strength.

To investigate the cellular interaction network in IDD, the R package “Cellchat” was performed. Compared to the control group, the number and the strength of interactions between different cell types were elevated in the IDD group (**Figure 5A**). Besides, we showed the relationship of the number of interactions between different cell types in the IDD and the control group. Relatively little difference was found between the groups (**Figure 5B**). Then, we focused on monocytes and found that the biggest change occurred between monocytes and macrophages. And the communication strength between the two cells decreased greatly in IDD, indicating that the interaction between macrophages and monocytes is an important factor in the occurrence of IDD (**Figure 5C**).

**Figure 6:**
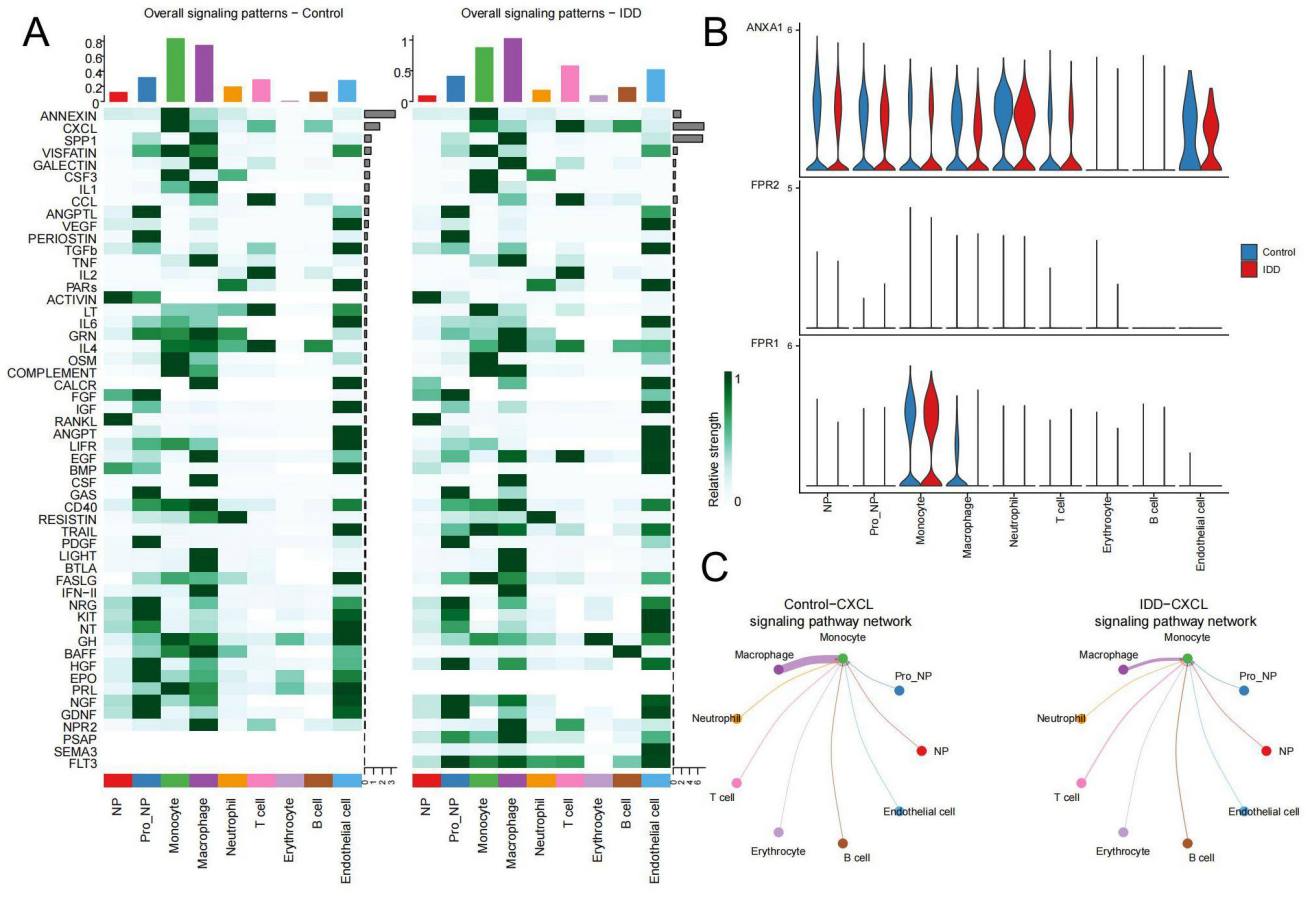
Changes in pathways during cellular communication **(A)** The heatmap displays the status of various pathways in cell communication between the control and IDD groups. The top bar chart reflects the total communication signals of all pathways within the corresponding cell types, while the right bar chart shows the total communication signals of each pathway across all cell types. The left panel represents the control group, and the right panel represents IDD. **(B)** The expression distribution of receptors in the ANNEXIN signaling pathway in both the control and IDD groups. **(C)** The network diagram illustrates interactions between the CXCL signaling pathway originating from Monocytes and other cell types.

Next, we visualized the relationship between immune cells and signaling pathways in the control and IDD group in the heatmap. It demonstrated that in the control group, ANNEXIN is the most important signaling pathway, but its communication signal decreases significantly after the occurrence of IDD (**Figure 6A**). To explore the reasons for this trend, violin plots were used to show the expression of the ANNEXIN pathway between the control and IDD groups. Compared with the control group, the ligand ANXA1 showed a decreasing trend in all cell types. And monocytes, with the highest expression of receptor FPR1, may be affected (**Figure 6B**). In addition, we also observed a significant decline in the CXCL signaling pathway in monocytes after the occurrence of IDD(**Figure 6A**). Thus, the CXCL pathway-mediated communication between monocytes and other cells was mapped. The results showed that the CXCL pathway of monocytes mainly occurred in communication with macrophages and was significantly reduced in IDD (**Figure 6C**). Therefore, the reduction of CXCL signaling in monocytes and macrophages may exacerbate the progression of IDD.

### 3.5 Identification of DEGs between IDD and control samples

**Figure 7:**
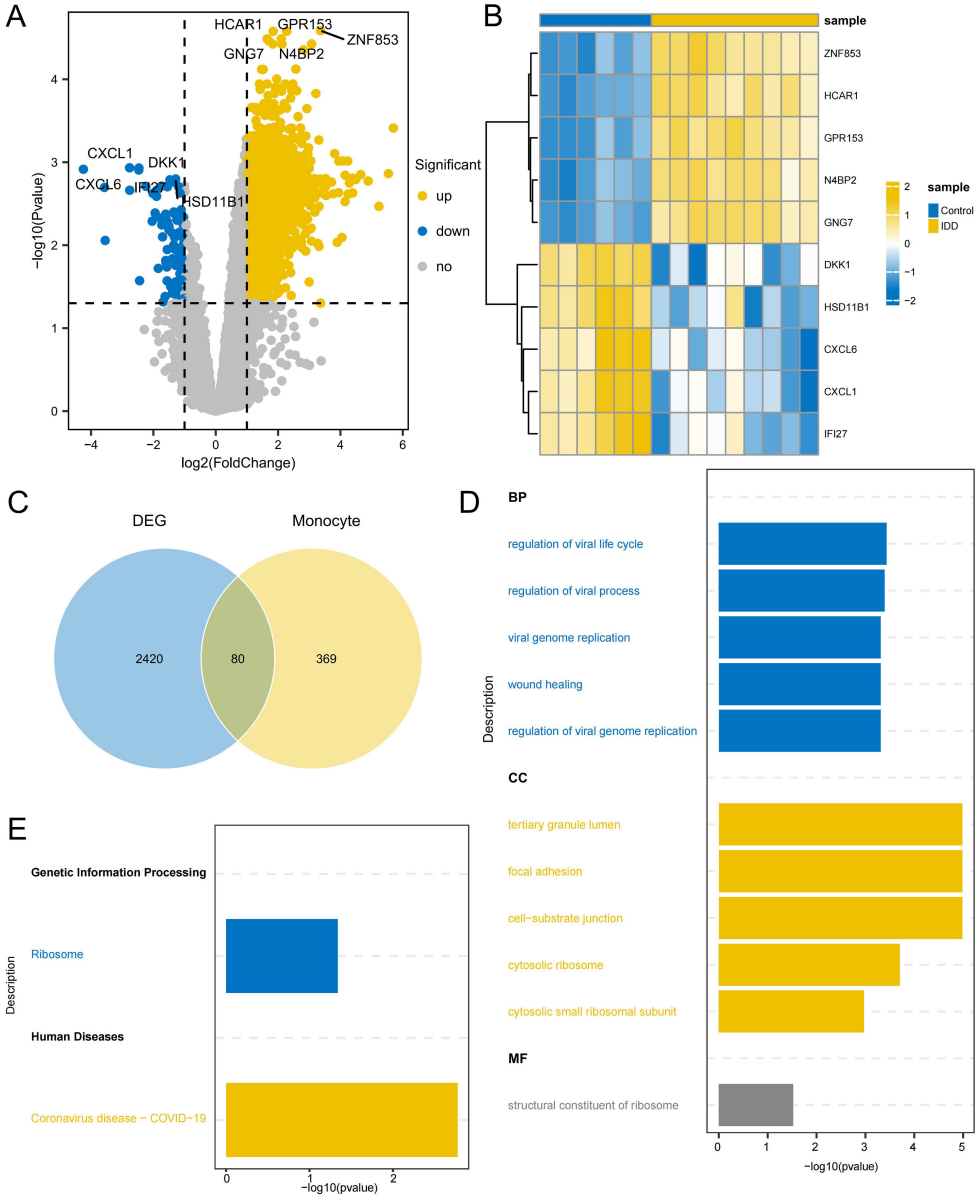
Identification of DEGs related to monocytes **(A)** Volcano plot depicting significant DEGs between IDD and control group **(B)** Heatmap of the top 5 DEGs with the highest significance in up and down-regulation **(C)** The Venn diagram illustrates the identification of key genes involved in regulating the occurrence of IDD in monocytes. **(D)** GO enrichment of the intersection DEGs, displaying the top 5 most significant pathways in Biological process (BP), Cellular component (CC), and Molecular function (MF). **(E)** KEGG enrichment of the intersection DEGs.

2,500 DEGs were identified between IDD and control group, including 2,413 up-regulated genes and 87 down-regulated genes(**Figures 7A, B**; **Supplementary Table 6**). By intersecting DEGs from the conventional transcriptome with DEGs from monocytes, 80 intersecting DEGs were identified as key genes (**Figure 7C**; **Supplementary Table 7**). GO enrichment analysis indicated that these genes play significant roles in BPs, such as regulation of the viral life cycle, regulation of viral processes, and viral genome replication. Additionally, CC, like tertiary granule lumen, focal adhesion, and cell-substrate junction, were enriched, as well as MF, such as the structural constituent of the ribosome (**Figure 7D**). KEGG analysis showed they were significantly enriched in Ribosome and Coronavirus disease - COVID-19 (**Figure 7E**; **Supplementary Table 8**). This suggests that ribosomes may be indirectly involved in the regulation of degenerative inflammatory responses by supporting the protein synthesis function of monocytes. The inflammatory environment of IDD activates monocytes, which synthesize and secrete a variety of inflammatory factors and degrading enzymes with the help of ribosomes, accelerating the destruction and degeneration of the intervertebral disc. Enrichment in viral biology suggests a role for the activation of inflammatory stress pathways in viral infection-mediated monocytes in IDD progression. Certain viruses, such as herpes simplex virus, can infect disc cells, causing direct cytopathy, inflammation, or apoptosis, which can accelerate disc degeneration (39). Coronavirus may also have a similar effect on disc degeneration.

### 3.6 Screening and validation of key genes

**Figure 8:**
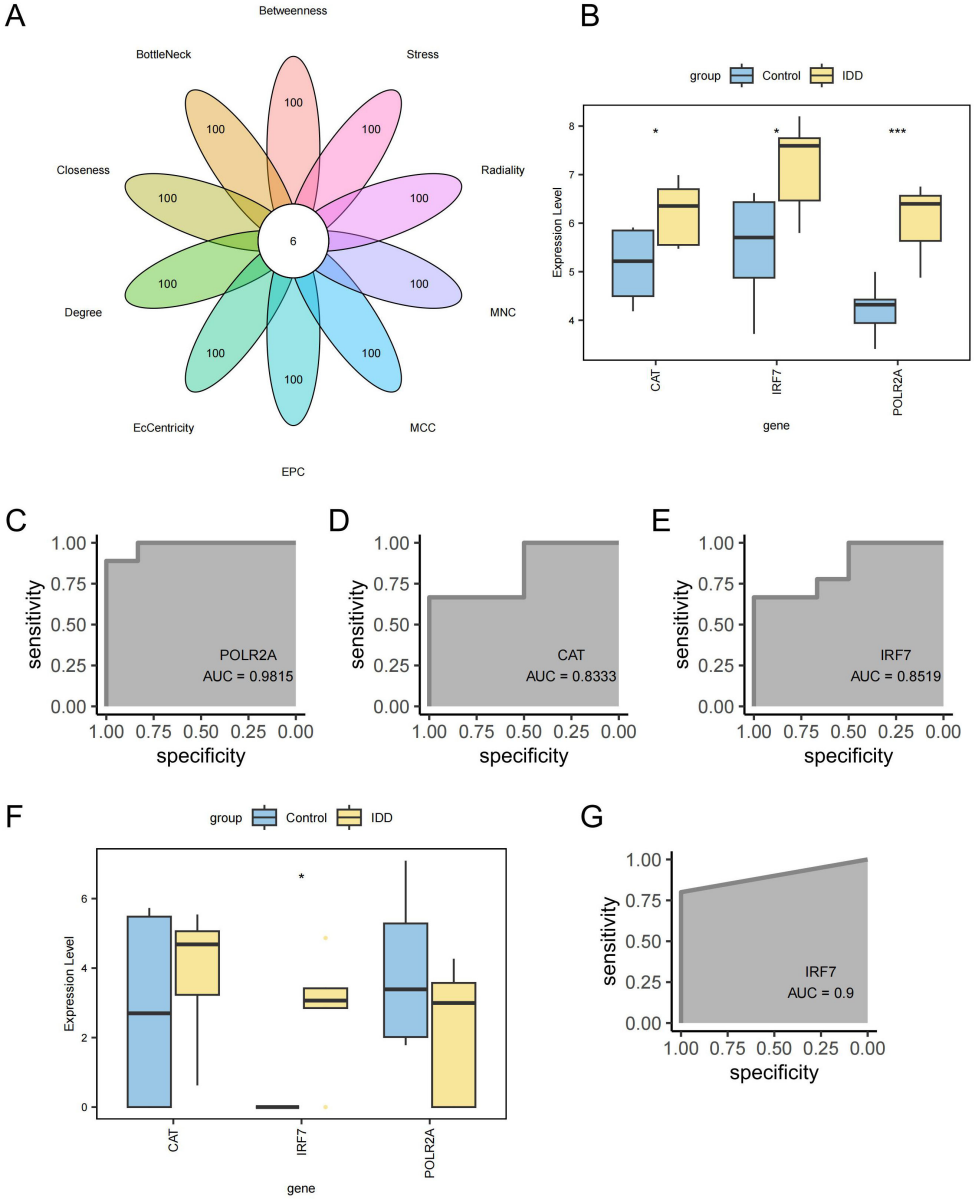
Screening of key Genes. **(A)** Six genes were identified through a combination of 10 network topology algorithms. **(B)** Key genes were significantly up-regulated in the IDD group. **(C)** ROC curve for gene POLR2A. **(D)** ROC curve for gene CAT. **(E)** ROC curve for gene IRF7. **(F)** Box plot showing differential expression of key genes in the external validation cohort GSE167931. **(G)** ROC curve for gene IRF7 in the external validation cohort GSE167931. ***p < 0.001, *p < 0.05.

A PPI network was created for the intersecting DEGs using the STRING online database. The top 100 proteins in terms of importance were screened using 10 algorithms, including Betweenness, BottleNeck, Closeness, Degree, EcCentricity, EPC, MCC, MNC, Radiality, and Stress. Six genes were identified through the intersection of these algorithms: POLR2A, RPLP0, JUN, CAT, IRF7, and RPS3 (**Figure 8A**). POLR2A, CAT, and IRF7 were included in the list of intersected DEGs, and RPLP0, JUN, and RPS3 were the genes that had a direct reciprocal relationship with the intersected DEGs. POLR2A, CAT, and IRF7 were selected as key genes for subsequent analysis. These essential genes showed up-regulation in the IDD group (**Figure 8B**). The ROC curves demonstrated POLR2A, CAT, and IRF7 all showed good efficacy for distinguishing IDD and standard samples (**Figures 8C–E**). Furthermore, the expression levels of these pivotal genes were confirmed in GSE167931. IRF7 were also significantly up-regulated. The expression of CAT in the IDD group is slightly upregulated, consistent with the trend in our analysis, which also shows potential as an IDD biomarker (**Figure 8F**). IRF7 showed promising efficacy for IDD and standard samples (AUC=0.9, **Figure 8G**).

### 3.7 Signaling pathways of key genes

**Figure 9:**
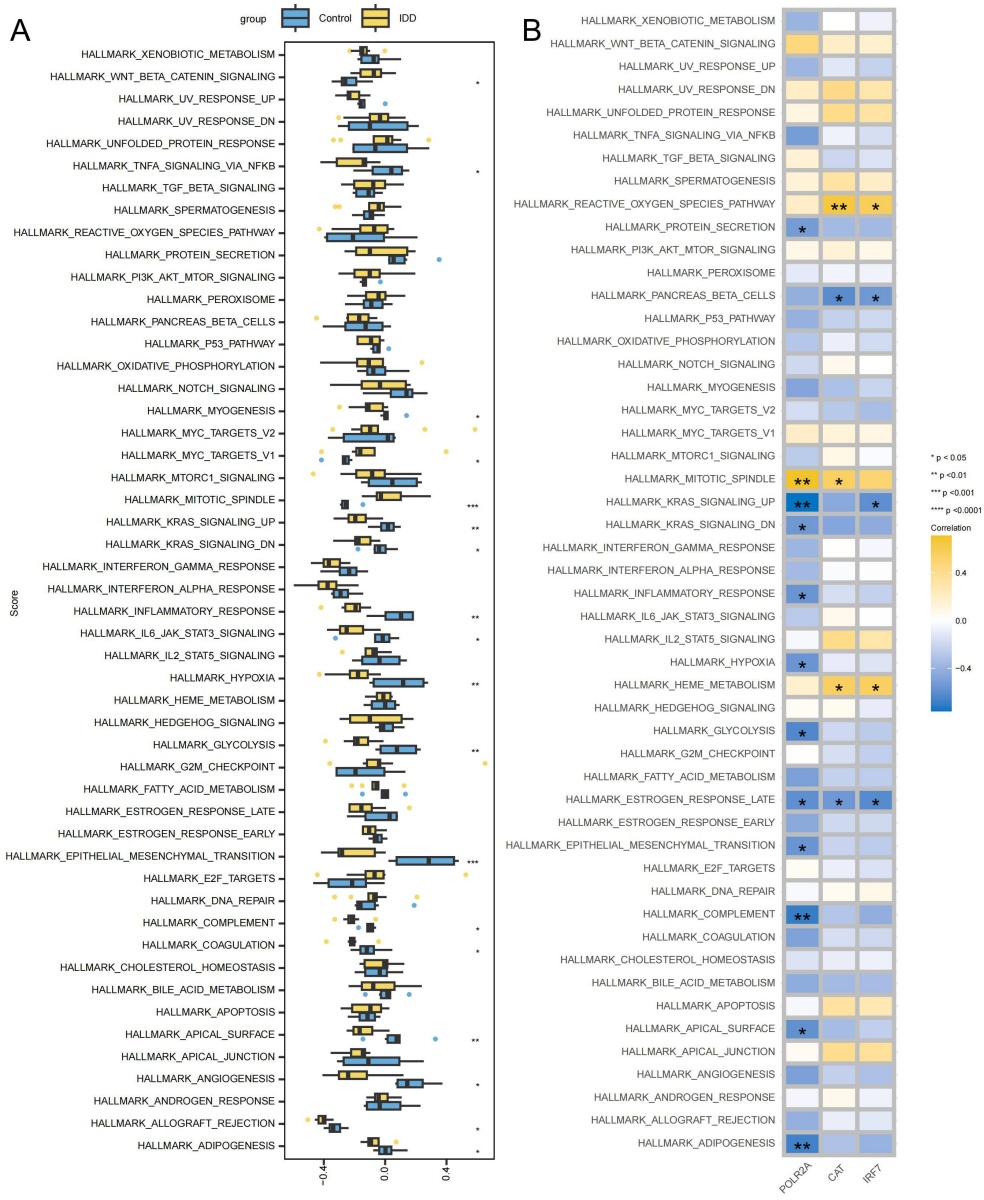
Signaling pathways associated with key genes **(A)** Box plot displaying differentially enriched pathways between the IDD and the control group. **(B)** Correlation between key genes and signaling pathways, with yellow indicating positive correlations and blue indicating negative correlations. ***p < 0.001, **p < 0.01, *p < 0.05.

GSVA showed the differences between the IDD and control groups across 50 Hallmark signaling pathways (**Supplementary Table 9**). In the IDD group, 3 pathways showed significant up-regulation and 15 pathways showed significant down-regulation (**Figure 9A**). IRF7 exhibits a significant negative correlation with HALLMARK_KRAS_SIGNALING_UP_PATHWAY, and down-regulation of HALLMARK_KRAS_SIGNALING_UP_PATHWAY has also been observed in IDD, suggesting that IRF7’s promotion of IDD may be mediated by inhibition of the KRAS pathway (**Figure 9B**). The activation of KRAS can upregulate the expression of the anti-apoptotic protein Bcl-2, thereby inhibiting apoptosis (40). In IDD, KRAS may be inhibited, leading to increased apoptosis of NPCs.

### 3.8 Construction of key gene co-expression networks

**Figure 10:**
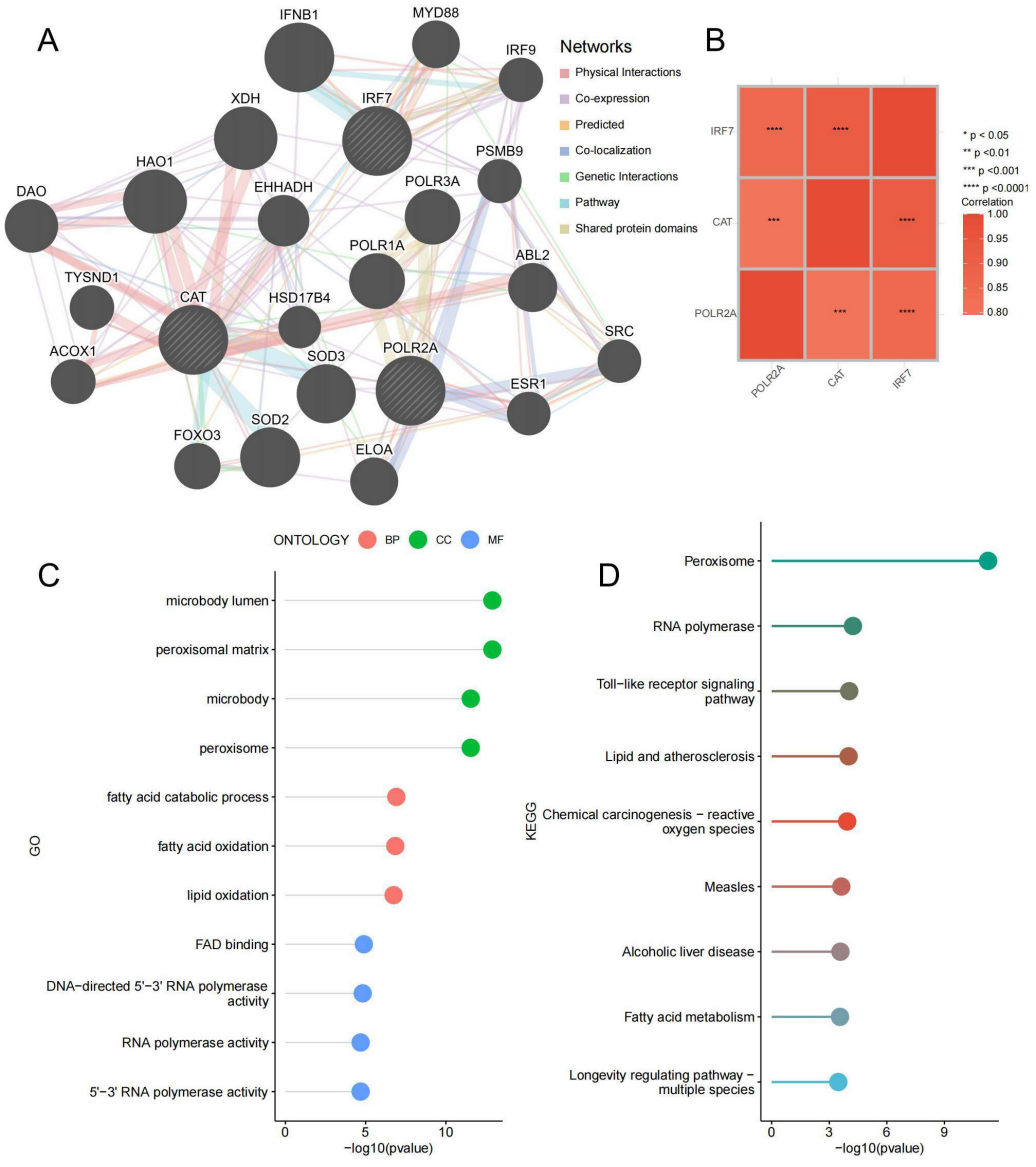
Co-expressed Gene Analysis. **(A)** Gene co-expression network plot, the size of the circles represents the overall interaction strength of the corresponding proteins within the network, while the thickness of the lines indicates the strength of interactions between pairs of proteins. **(B)** Heatmap of correlations between key genes. **(C)** GO enrichment lollipop plot of co-expressed genes. **(D)** KEGG enrichment lollipop plot of co-expressed genes. ***p < 0.001, ****p < 0.0001.

To delve deeper into the function of the key genes, a co-expression network was constructed utilizing the GeneMANIA database (**Figure 10A**; **Supplementary Table 10**). The correlation heatmap between hub genes is shown in **Figure 10B**, demonstrating that most key genes were closely correlated with each other. GO and KEGG enrichment analysis found that it is related to lipid metabolism and peroxide pathways. This suggests that three hub genes and closely interacting genes may be involved in IDD progression by mediating these processes (**Figures 10C, D**; **Supplementary Table 11**). Specifically, lipid metabolism disorders can lead to abnormal deposition of lipids in the intervertebral discs, triggering an inflammatory response (41). Lipid peroxidation accelerates disc cell damage and matrix degradation by increasing oxidative stress and promoting an inflammatory response (42). These processes interact with each other to form a vicious circle that ultimately leads to the progression of disc degeneration.

### 3.9 Correlation network construction of key genes

**Figure 11:**
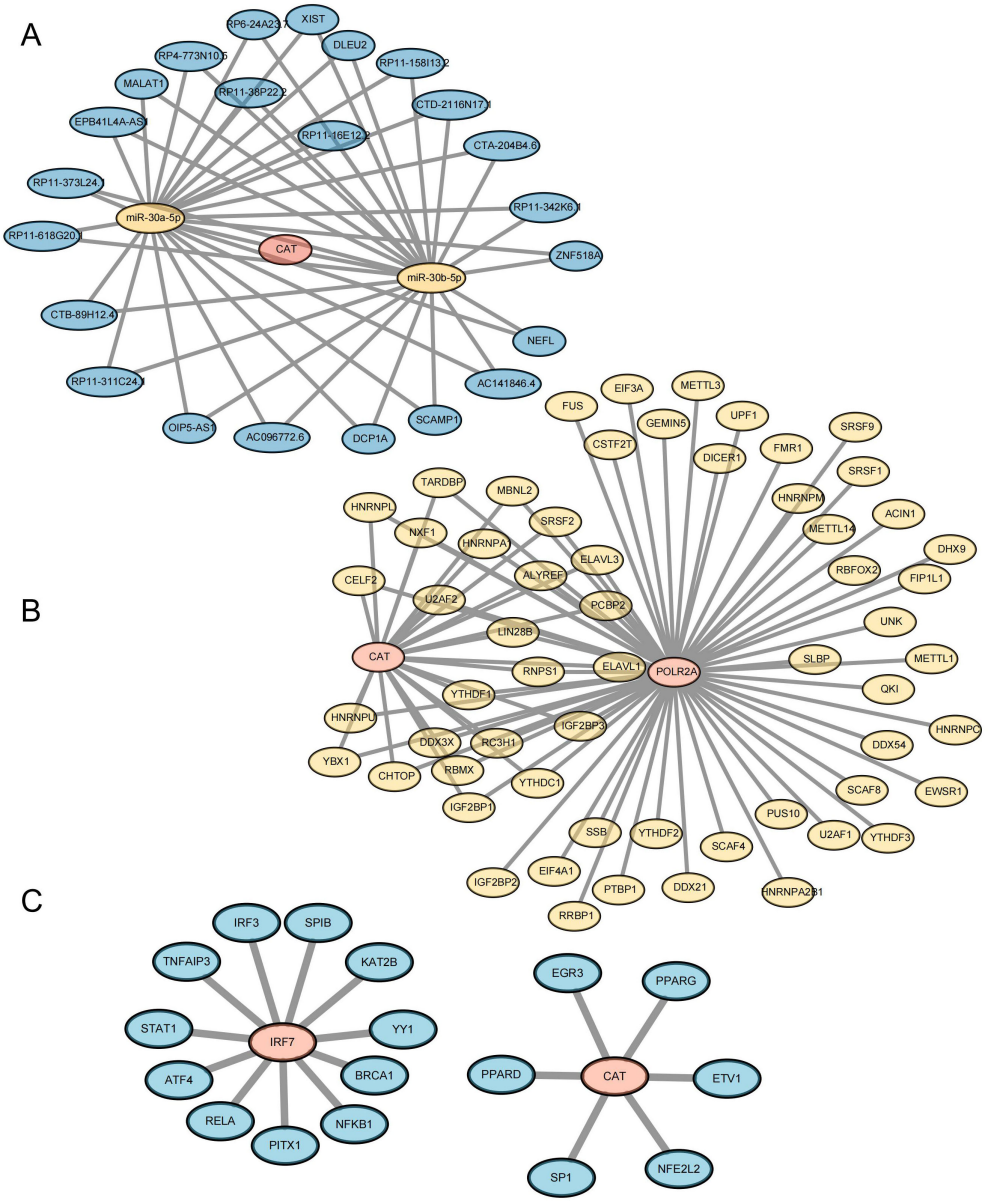
Construction of Key Gene-Related Networks. **(A)** lncRNA–miRNA–mRNA network of key genes. Blue represents lncRNA, yellow represents miRNA, and pink represents mRNA. **(B)** RBP-mRNA regulatory network of key genes. Yellow represents RBP, and pink represents mRNA. **(C)** mRNA-TF interaction network of key genes. Pink represents target genes; blue represents transcription factors (TF).

In order to further study the way of hub gene regulation, we constructed mRNA, TF, and ceRNA regulatory networks. An mRNA-miRNA-lncRNA interaction network was constructed using the key genes. Two miRNAs (miR-30a-5p and miR-30b-5p) were found to bind to CAT. A total of 23 lncRNAs were identified as target lncRNAs. The ceRNA network is shown in **Figure 11A** (**Supplementary Table 12**). The corresponding mRNA/RBP pairs of hub mRNAs with pairwise information were searched and downloaded using the StarBase online database. Based on the relationships identified among the target genes from the online dataset, we constructed an intricate RBP-mRNA network (**Figure 11B**; **Supplementary Table 13**). Transcription factors (TFs) that bind to co-expressed genes were searched using the TRRUST database, identifying interaction relationship data for 2 target genes and 17 TFs (**Figure 11C**; **Supplementary Table 14**).

### 3.10 Differential expression of IRF7 was the most significant, and knockdown of IRF7 alleviated IDD in NPCs

**Figure 12:**
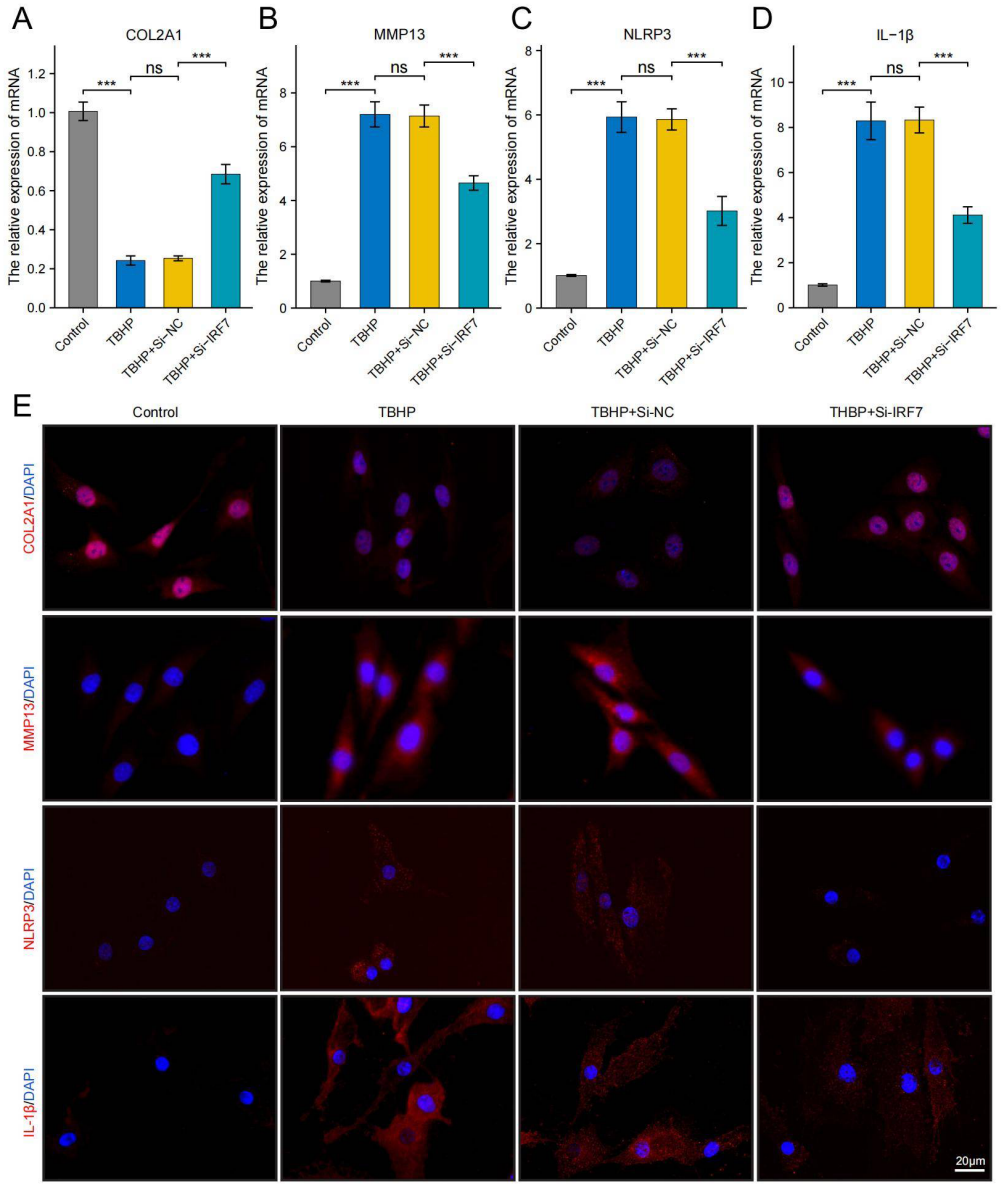
Knockdown of the IRF7 gene can alleviate the degeneration of NPCs. **(A–D)** RT-qPCR shows changes in COL2A1, MMP13, NLRP3 and IL-1β expression levels. **(E)** Immunofluorescence shows changes in COL2A1, MMP13, NLRP3 and IL-1β expression levels.(scale bar: 20μm) The data were presented as the mean ± SD. ***p < 0.001.

Using RT-qPCR analysis, it was noted that IRF7, POLR2A, and CAT exhibited significant upregulation in the TBHP-induced degeneration group, with IRF7 showing the most significant increase in expression (**Supplementary Figure 1**). IDD can cause an imbalance between catabolism and anabolism in the ECM, leading to structural changes in the intervertebral disk (43). To investigate the potential of knocking down IRF7 to reverse IDD *in vitro*, we set up four groups of experiments: standard group, IDD group (TBHP), Si-NC group (TBHP + Si-NC), and Si-IRF7 group (TBHP + Si-IRF7). The efficiency of the IRF7 knockdown in NPCs was verified by RT-qPCR (**Supplementary Figure 1**). It was found that knocking down IRF7 increased the expression of COL2A1 in the Si-IRF7 group while decreasing the expression of MMP13 compared to the Si-NC group (**Figures 12A, B, E**). It indicates that the knockdown of IRF7 can reverse the metabolic imbalance of the extracellular matrix (ECM). Besides,the knockdown of IRF7 decreased the expression of NLRP3 and IL-1β compared to the Si-NC group (**Figures 12C–E**). This indicates that knocking down IRF7 can alleviate the inflammation of IDD. In conclusion, these findings demonstrate that the knockdown of IRF7 can slow the progression of NPC degeneration.

### 3.11 *In vivo* therapeutic effects of knocking down IRF7 in a rat model of IDD

**Figure 13:**
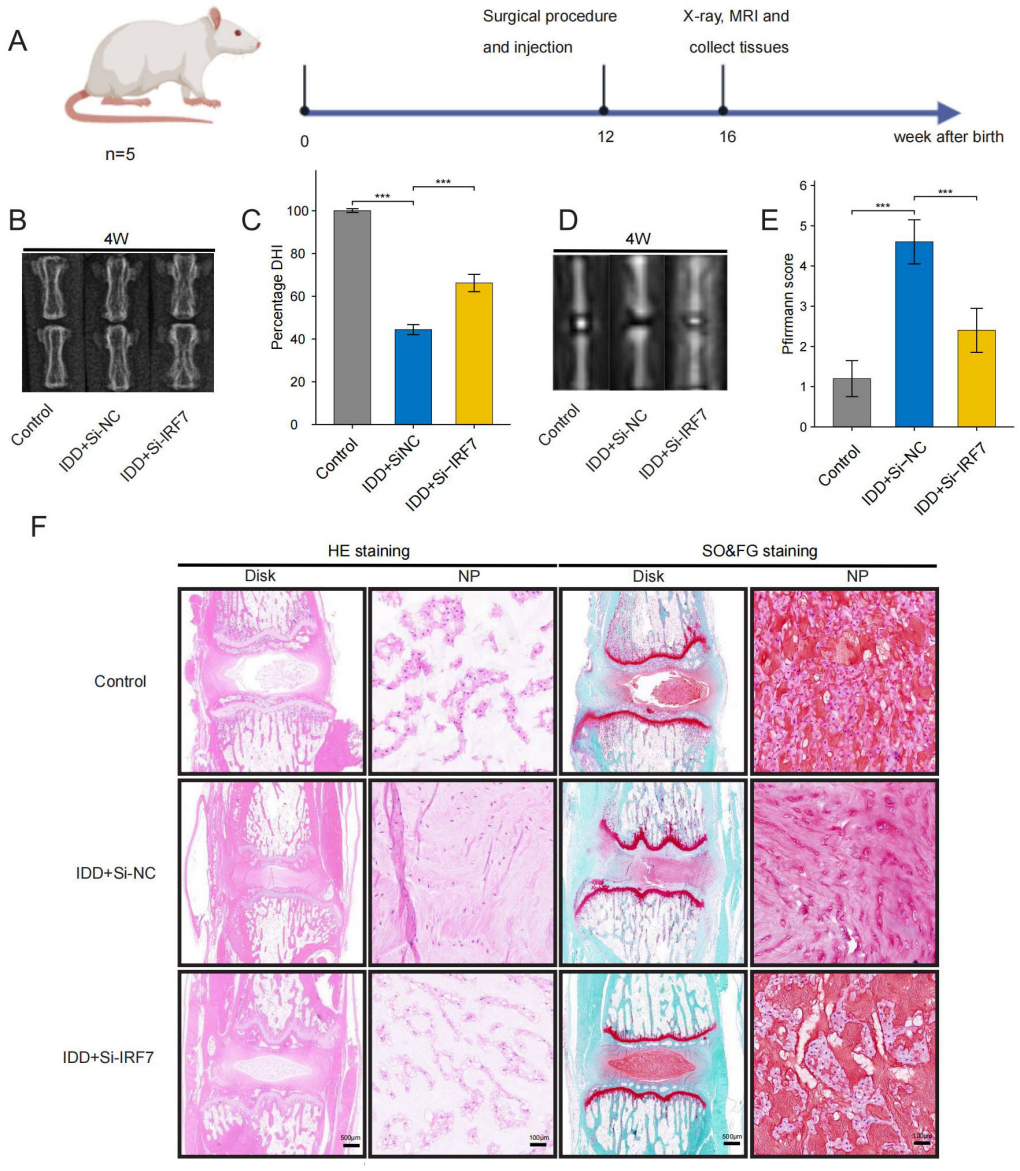
Therapeutic effects of IRF7 knockdown in rats. **(A)** Flowchart of the animal experiment **(B, C)**. X-ray images and DHI scores 4 weeks after surgery **(D, E)**. T2-weighted MRI images and Pfirrmann grading scores 4 weeks after surgery; **(F)**. Representative images of HE and SO&FG staining at 4 weeks after surgery. (original images, scale bar: 500 μm; enlarged images, scale bar: 100μm) The data were presented as the mean ± SD. ***p < 0.001.

**Figure 14:**
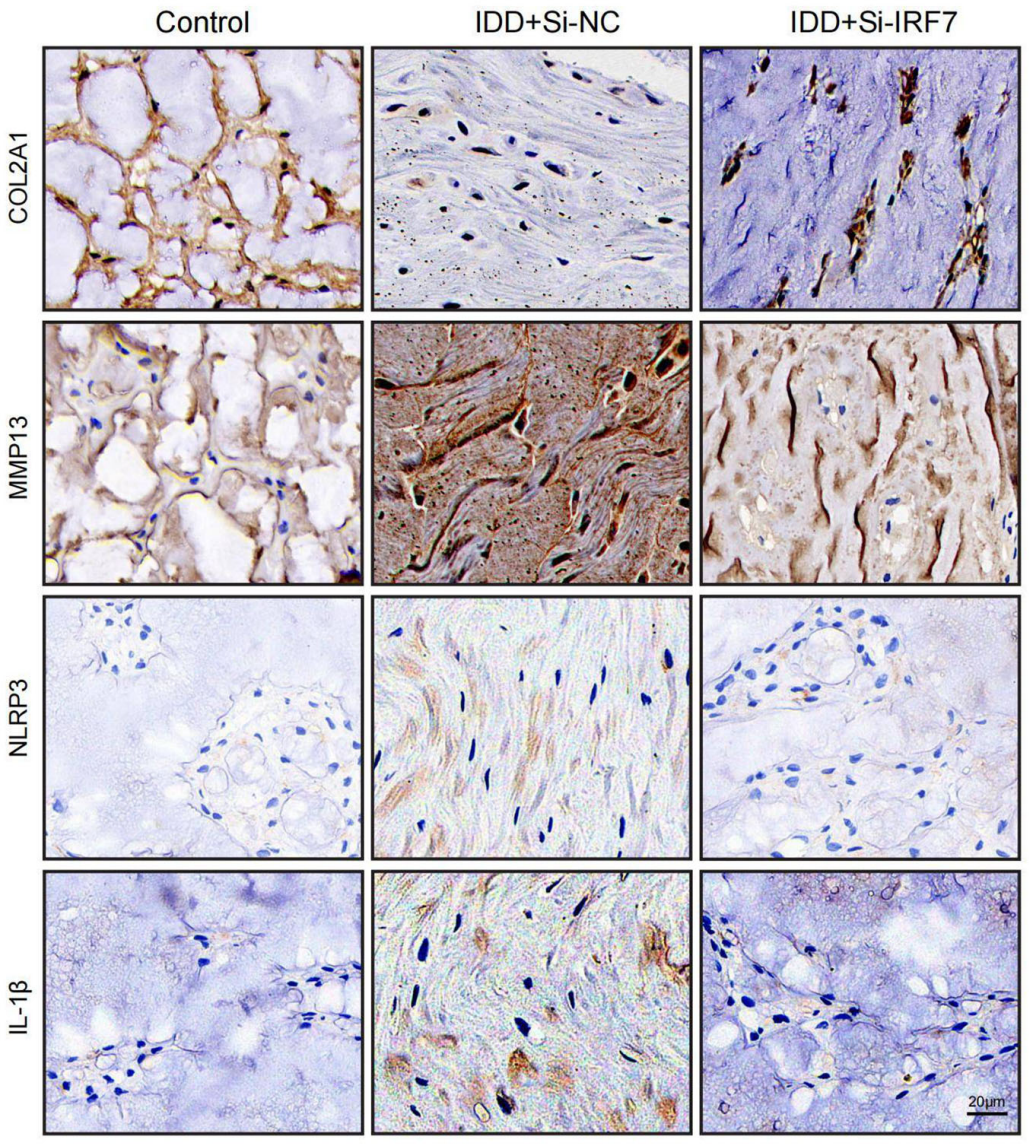
Representative images of immunohistochemistry at 4 weeks after surgery (scale bar: 20μm).

Finally, we investigated the potential of knocking down IRF7 to reverse IDD *in vivo*. The flowchart of the animal experiment is shown in **Figure 13A**. Immunohistochemistry showed higher IRF7 expression in the Si-NC group than in the control group, and IRF7 was successfully knocked down *in vivo* (**Supplementary Figure 2**). 4 weeks after the knockdown of IRF7, X-ray analysis showed an increased intervertebral space height in the Si-IRF7 group compared to the Si-NC group, with corresponding increases in DHI scores (**Figures 13B, C**). MRI results indicated significantly higher signal intensity in the Si-IRF7 group than in the Si-NC group, consistent with the grading results (**Figures 13D, E**). Both X-ray and MRI analyses confirmed that the knockdown of IRF7 alleviated IDD. Histological analysis further validated the therapeutic effects of IRF7 knockdown. It was found that NPs in the Si-NC group gradually shrank with blurred borders and were replaced by AF contents. In contrast, the NPs in the Si-IRF7 group showed significantly restored morphology (**Figure 13F**). The expression of COL2A1, MMP13, NLRP3 and IL-1β in NP tissues was accessed with immunohistochemical staining. The knockdown of IRF7 significantly restored the expression of COL2A1, while the expression of MMP13, NLRP3 and IL-1β was significantly reduced (**Figure 14**). These findings collectively demonstrate that the knockdown of IRF7 can inhibit inflammation and reverse the metabolic imbalance of the ECM, thereby alleviating the progression of IDD.

### 3.12 Forecast of potential therapeutic drugs for IDD

**Figure 15:**
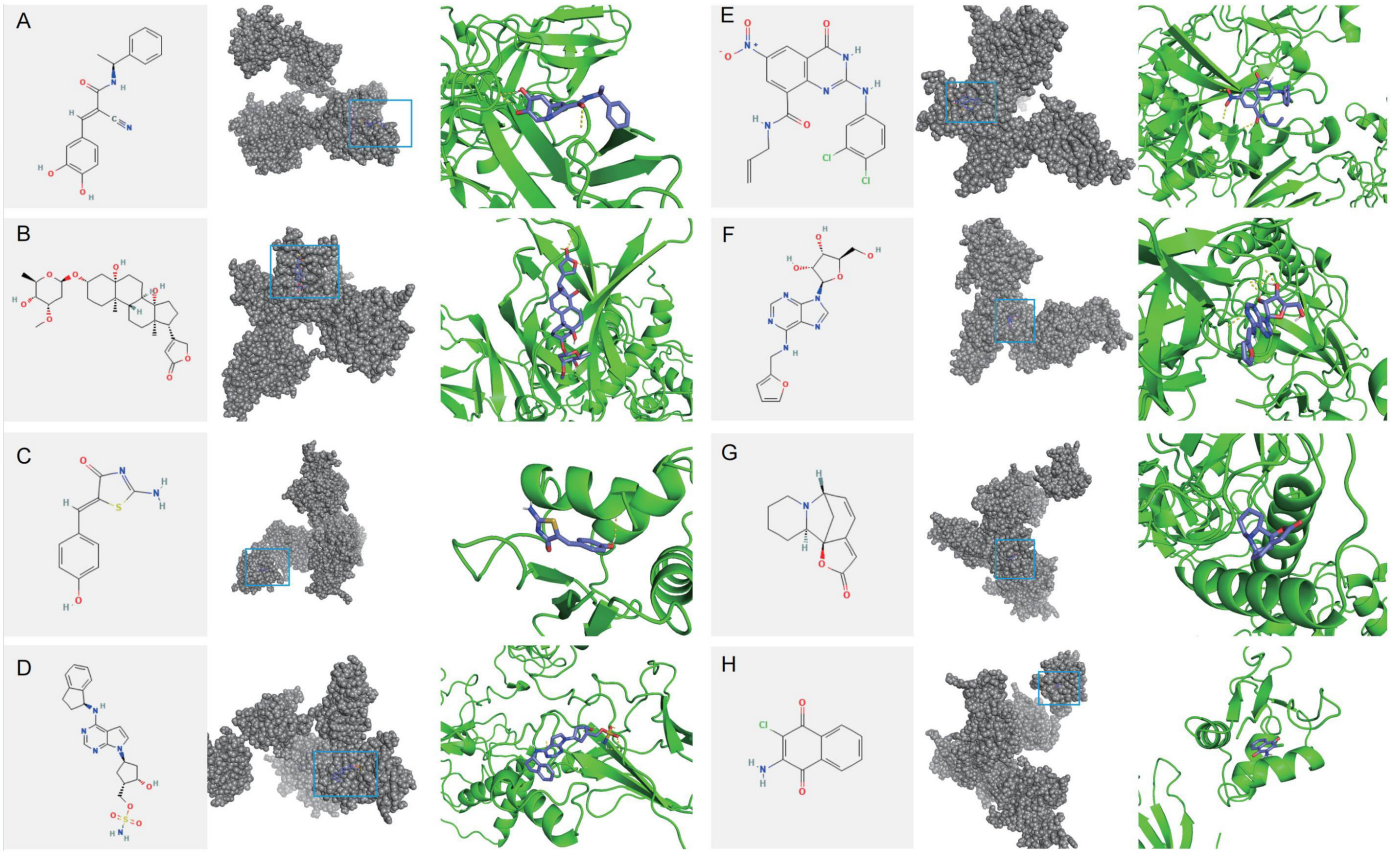
Molecular docking of potential drug molecules with their corresponding target proteins. **(A)** Tyrphostin AG 835 **(B)** Periplocymarin **(C)** Mirin **(D)** MLN-4924 **(E)** AZ-10417808 **(F)** Kinetin riboside **(G)** Securinine **(H)** Quinoclamine.

To identify potential therapeutic drugs for IDD, 10 small molecule compounds most likely to serve as potential drugs were predicted based on the CMAP database (**Supplementary Table 15**). Homoharringtonine, which exhibits intense toxicity, was excluded from the analysis as a potential drug. Additionally, the structure of QW-BI-011 could not be found. Therefore, eight small-molecule compounds were identified as potential drugs: Tyrphostin-AG-835, Periplocymarin, Mirin, MLN-4924, AZ-10417808, Kinetin-riboside, Securinine, and Quinoclamine. Subsequently, molecular docking analysis of potential small-molecule drugs with IRF7 was performed. The molecular docking demonstrated that the binding energies of IRF7 with these potential small molecule drugs were less than -3 kcal/mol, indicating strong binding affinity. This suggests that these small-molecule drugs may exert therapeutic effects by binding to IRF7 for IDD treatment (**Figure 15**).

## 4 Discussion

LBP is a severe medical and social problem worldwide. It is also a cause of total disability in middle-aged and older adults and the most common reason for activity limitation in patients under 45 years of age (44–46). IDD is the leading cause of LBP. A growing body of research suggests that in addition to biomechanical factors, the autoimmune system plays an essential role in the degenerative process of this disease, including immune cells and cytokines (1, 47). When the AF or CEP suffers damage or degeneration, it can lead to the infiltration of immune cells into the intervertebral disc. This migration leads to inflammation within the disc, resulting in increased cytokine and chemokine levels. This vicious cycle of inflammation-driven catabolism accelerates ECM breakdown (17, 43). Therefore, the treatment strategy for IDD should no longer be limited to traditional physical therapy, surgical intervention, and pharmacological analgesia but gradually shift to modulating the immune response, suppressing the inflammatory response, and promoting intervertebral disc repair. These emerging therapies are expected to provide patients with IDD with more effective and lasting solutions to reduce LBP symptoms and improve quality of life.

Monocytes are a type of blood cell originating from bone marrow precursors and associated with the mononuclear phagocytic cell system (48). Monocytes are the second-line defense cells of the innate immune system after neutrophils and can engulf foreign particles. They can differentiate into their subpopulations and macrophages depending on the necessities of the microenvironment. In addition, monocytes produce cytokines and act as antigen-presenting cells (APCs) (49). In this study, machine-learning techniques were used to screen for immune cells, identifying monocytes as key players in the development and progression of IDD. Consistent with our findings, Guo et al. found through bioinformatics analysis that monocytes infiltration was more frequent in the IDD group compared to the healthy group (50). Inflammation is a crucial factor in IDD progression, and IL-17 has been shown to recruit monocytes and neutrophils to sites of inflammation by increasing chemokine production (43). It can further amplify the inflammatory cascade, promote the degradation of the ECM, and accelerate the progression of IDD. As immune cells, monocytes initiate the defense against inflammation upon recruitment to inflammation sites under the chemotaxis of cytokines (51). A recent study also found that monocytes are abnormally activated in the late stages of IDD (52). Therefore, monocytes may play a pivotal role in the progression of IDD, leading to increased inflammation within the intervertebral disc.

The interaction of cytokines with intervertebral disc cells is one of the crucial mechanisms of IDD. NPCs undergo a series of biological responses stimulated by cytokines, including apoptosis, proliferation, and matrix degradation (53). For example, IL-1β and TNF-α can promote the secretion of more inflammatory factors and matrix-degrading enzymes from NP and AF cells by activating the NF-κB and MAPK signaling pathways, thus forming a vicious circle and accelerating IDD (54). These cytokines promote the inflammatory response and lead to the degradation of the ECM by regulating the expression of enzymes such as matrix metalloproteinases (MMPs) and ADAMTS, thus accelerating IDD (55). In addition, it has been found that specific anti-inflammatory cytokines, such as IL-10, are expressed at low levels in intervertebral disc tissues, which may contribute to uncontrolled inflammation and increased degeneration (56). To systematically elucidate the effects of cytokines on monocytes in IDD, the present study identified an important role of type I interferon(IFN-a1, IFN-b) in the monocytes by IREA analysis. Type I interferons play a crucial role in antiviral immunity and influence inflammatory response and tissue repair by modulating the function of multiple immune cells. They regulate the innate immune response by promoting antigen presentation and natural killer cell function (57). Besides, they can modulate inflammation by affecting key factors in various signaling pathways such as the JAK/STAT pathway, TLRs pathway, NF-κB pathway, PI3K/AKT pathway, and MAPK pathway (58). Secondly, IFN-Is can trigger the adaptive immune system and promote the development of high-affinity antigenic immune cell responses and immune memory (59, 60). During disc degeneration, monocytes may increase type I interferons, causing monocytes mono-a polarization, increasing oxidative stress and promoting inflammatory responses to accelerate disc cell damage and matrix degradation.

Cell-cell communication analysis in IDD revealed that the ANNEXIN pathway was inhibited in the IDD group. Annexins were described as Ca^2+^-regulated membrane-binding modules that respond to cellular stress and control mammal inflammatory responses (61). Annexins have been proven to play an anti-inflammatory role in various diseases. Jia et al. found that Annexin A5 inhibited the release of pro-inflammatory mediators *in vitro* and reduced the production of reactive oxygen species in osteoarthritis, which protected chondrocyte necrosis and apoptosis (62). Similarly, You et al. found that Annexin A5 depletion exacerbates vascular remodelling and dysfunction while upregulating the expression of age- and inflammation-related proteins (63). Thus, the inhibition of the ANNEXIN pathway in IDD may prevent it from responding adequately to inflammatory stimuli in the intervertebral disc, contributing to the progression of IDD. As a member of the Annexin family, Annexin A1 exerts anti-inflammatory and pro-catabolic effects in various ways (64). In our study, we also found that the ligand ANXA1 showed a downward trend in all cell types in IDD, which may lead to a decrease in the anti-inflammatory effect of the IDD group. Michael Scannell found that AnxA1 can induce monocytes recruitment and enhance phagocytosis, confirming our conjecture (65). Additionally, Shao et al. found that NPCs can seek the help of neutrophils through the ANXA1-FPR1 pathway to alleviate intervertebral disc inflammation (66). Our study found that the receptor FPR1 expression was highest among monocytes. Therefore, the ANXA1-FRP1 pathway may alleviate inflammation in the intervertebral disc by recruiting monocytes.

Subsequently, we intersected the genes specifically expressed in monocytes from the single-cell data with the DEGs between IDD and healthy controls from the transcriptome. The core genes POLR2A, CAT, and IRF7 were identified using ten algorithms. The differential expression of CAT and IRF7 was validated in an external dataset, GSE167931, further confirming their central roles in IDD development. Similar results were obtained from *in vitro* experiments, where significantly higher expression of POLR2A, CAT, and IRF7 was observed in TBHP-induced IDD samples, verifying the reliability of our analysis.

RNA polymerase II subunit A (POLR2A) is the largest subunit encoding RNA polymerase II, which plays a crucial role in transcription. Although no literature directly supports its relationship with IDD, external validation has demonstrated high expression of POLR2A in the IDD group. GSVA supported the significant correlation between POLR2A and numerous Hallmark pathways enriched in the IDD group, indicating that abnormalities in POLR2A can lead to changes in multiple pathways, such as HALLMARK_MITOTIC_SPINDLE and HALLMARK_KARS_SIGNALING_UP, thereby affecting IDD progression. CAT encodes catalase, a critical antioxidant enzyme that protects cells from oxidative damage. Xiang et al. found that Nrf2 signaling promotes the transcription of downstream antioxidant genes, including CAT, which in turn come to protect against oxidative stress in intervertebral disc cells (67). Xiao et al. found that CAT could significantly reduce oxidative stress in intervertebral disc cells, thereby reducing apoptosis and inflammation and slowing down disc degeneration (68). GSVA analysis also showed that CAT was associated with HALLMARK_REACTIVE_OXYGEN_SPECIES_PATHWAY, further supporting its protective role in oxidative stress regulation. Thus, CAT may slow the progression of IDD by acting on the process of oxidative stress.

Interferon regulatory factor 7 (IRF7), a member of the IRF family, is a crucial regulator of type I interferon production (69). IRF7 can form a positive feedback loop with IFN-Is, significantly enhancing their expression and maintaining high levels of IFN-Is, thus actively regulating IFN-I production (70, 71). Koroth et al. found that interferons can play a crucial role in disc degeneration by modulating macrophage polarisation and influencing the inflammatory response of disc cells (72). Several studies have shown that IRF7 regulates inflammation in various diseases, however, its role in IDD remains unelucidated. Chen et al. demonstrated that IRF7 can promote apoptosis of intestinal epithelial cells and the release of pro-inflammatory proteins by activating the Nod-like receptor (NLR) pathway, thereby exacerbating intestinal inflammation (73). He et al. found that ILC2 from asthmatics exhibited much higher levels of IRF7 than healthy donors upon stimulation with papain or IL-33, suggesting that IRF7 may promote the development of asthma (74). Moreover, Aryl Hydrocarbon Receptor Interacting Protein (AIP) can inhibit IRF7 by antagonizing its nuclear localization, hindering IRF7-induced IFN-I production, reducing immune responses, and promoting aberrant inflammation (75).In this study, IRF7 was identified as a critical gene for IDD through PPI network analysis and was significantly up-regulated in IDD samples. *In vitro*, it was found that knocking down IRF7 can slow the progression of IDD. Compared with the Si-NC group, the expression levels of COL2A1 increased, while the expression level of MMP13, NLRP3 and IL-1β decreased after IRF7 knockdown. Subsequently, an IDD model in rats was constructed to verify the function of IRF7 further. X-ray showed that the intervertebral disc height in the Si-IRF7 group was higher than in the Si-NC group. MRI revealed that the water content of the intervertebral disc in the Si-IRF7 group was also higher than in the Si-NC group. HE and SF staining demonstrated that the morphology of the NP in the Si-IRF7 group was better restored than in the Si-NC group. Finally, immunohistochemistry confirmed that knocking down IRF7 can delay the progression of IDD by regulating inflammation and the metabolism of the extracellular matrix. Therefore, this study demonstrates through *in vivo* and *in vitro* experiments that IRF7 is an essential therapeutic target for IDD, and knocking down IRF7 can significantly alleviate IDD.

Eight small-molecule drugs were then screened that may alleviate IDD: Tyrphostin-AG-835, Periplocymarin, Mirin, MLN-4924, AZ-10417808, Kinetin-riboside, Securinine, and Quinoclamine. Molecular docking showed that IRF7 tightly binds to these potential small-molecule drugs, suggesting they may exert therapeutic effects by binding to IRF7 in IDD.

However, this study is subject to certain constraints. Firstly, the crucial role of monocytes and IRF7 in IDD has been demonstrated, but the specific mechanisms of action remain to be further investigated. Additionally, conducting *in vitro* drug tests and clinical trials is crucial to confirm the therapeutic effect of these small-molecule drugs on the progression of IDD.

## 5 Conclusion

Monocytes are essential in the progression of IDD, with the polarisation state of monocytes mediated by type I interferon potentially exacerbating the inflammatory response in the intervertebral disc. IRF7 has been identified as a key target for IDD, and its role in causing IDD has been confirmed through both *in vitro* and *in vivo* studies. These studies provide novel insights into potential therapeutic targets for IDD. Future research should encompass more extensive clinical sample analyses to confirm these discoveries and facilitate their translation into clinical applications.

## Data Availability

The original contributions presented in the study are included in the article/**Supplementary Material**. Further inquiries can be directed to the corresponding author.

## Supplementary material

Supplementary Table 1 Primers used in this study.

Supplementary Table 2 The result of ssGSEA.

Supplementary Table 3 DEGs in monocytes of IDD and Control groups.

Supplementary Table 4 The result of IREA(cytokines).

Supplementary Table 5 The result of IREA(cell polarisation).

Supplementary Table 6 2500 DEGs between IDD and control groups.

Supplementary Table 7 Intersecting 80 DEGs.

Supplementary Table 8 The results of the GO and KEGG enrichment analysis.

Supplementary Table 9 The results of GSVA.

Supplementary Table 10 The co-expression network of key genes.

Supplementary Table 11 GO and KEGG enrichment of 23 genes.

Supplementary Table 12 The results of the ceRNA network.

Supplementary Table 13 The results of the RBP network.

Supplementary Table 14 The results of the TF network.

Supplementary Table 15 The results of small-molecule drug prediction..

Supplementary Figure 1 Differential expression of three hub genes and IRF7 was knocked down *in vitro*.

Supplementary Figure 2 IRF7 was successfully knocked down *in vivo*.

## Glossary

IDD: Intervertebral disc degeneration
LBP: low back pain
AF: Annulus fibrosus
NP: Nucleus pulposus
CEP: Cartilaginous endplate
NPC: Nucleus pulposus cell
GEO: Gene Expression Omnibus
PCA: Principal component analysis
PC: Principal component
SNN: Shared Nearest Neighbor
t-SNE: t-distributed stochastic neighbour embedding
ssGSEA: Single sample gene set enrichment analysis
GSEA: Gene set enrichment analysis
SVM-RFE: Support Vector Machine-Recursive Feature Elimination
MDA: Mean decrease accuracy
MDG: Mean decrease Gini
IREA: Immune Response Enrichment Analysis
DEG: Differentially expressed genes
PPI: Protein-protein Interaction
STRING: Search Tool for the Retrieval of Interacting Genes
GO: Gene Ontology
KEGG: Kyoto Encyclopedia of Genes and Genomes
BP: Biological Process
MF: Molecular Function
CC: Cellular Component
ceRNA: Competing endogenous RNA
RBP: RNA-binding protein
TRRUST: Transcription Regulatory Relationships Unraveled Sentence-based Text Mining
FBS: Fetal bovine serum
siRNA: Small interfering RNA
SD: Sprague-Dawley
MRI: Magnetic resonance imaging
DHI: Disc height index
HE: Hematoxylin-eosin
SO&FG: Safranin O-Fast Green
BSA: Bovine serum albumin
Pro_NP: Progenitor nucleus pulposus
GSVA: Gene Set Variation Analysis
TF: Transcription factor
ECM: Extracellular matrix
CMAP: Connectivity Map
APC: Antigen-presenting cell
MMP: Matrix metalloproteinase
GC: Glucocorticoid
POLR2A: RNA polymerase II subunit A
IRF7: Interferon regulatory factor 7;NLR, Nod-like receptor
AIP: Aryl Hydrocarbon Receptor Interacting Protein.
UMAP: Uniform Manifold Approximation and Projection
NLR: Nod-like receptor
