# Supplementary figures and images for: “Dictionary of immune responses” reveals the critical role of monocytes and the core target IRF7 in intervertebral disc degeneration
Source: Front Immunol. 2024 Oct 17;15:1465126. doi: 10.3389/fimmu.2024.1465126 (PMC11524831; doi:10.3389/fimmu.2024.1465126)

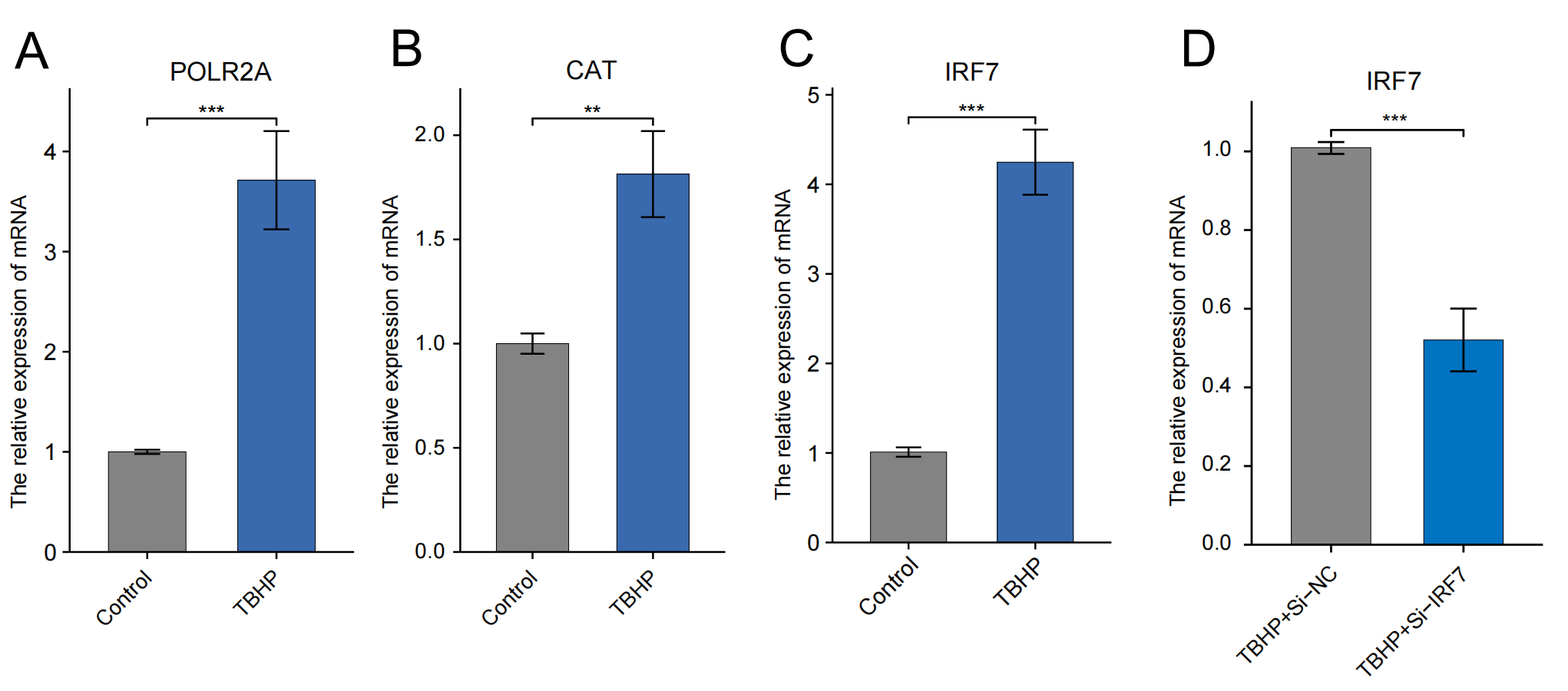

Supplement: Supplementary Figure 1 — Differential expression of three hub genes and IRF7 was knocked down in vitro. [file Image1.jpeg]

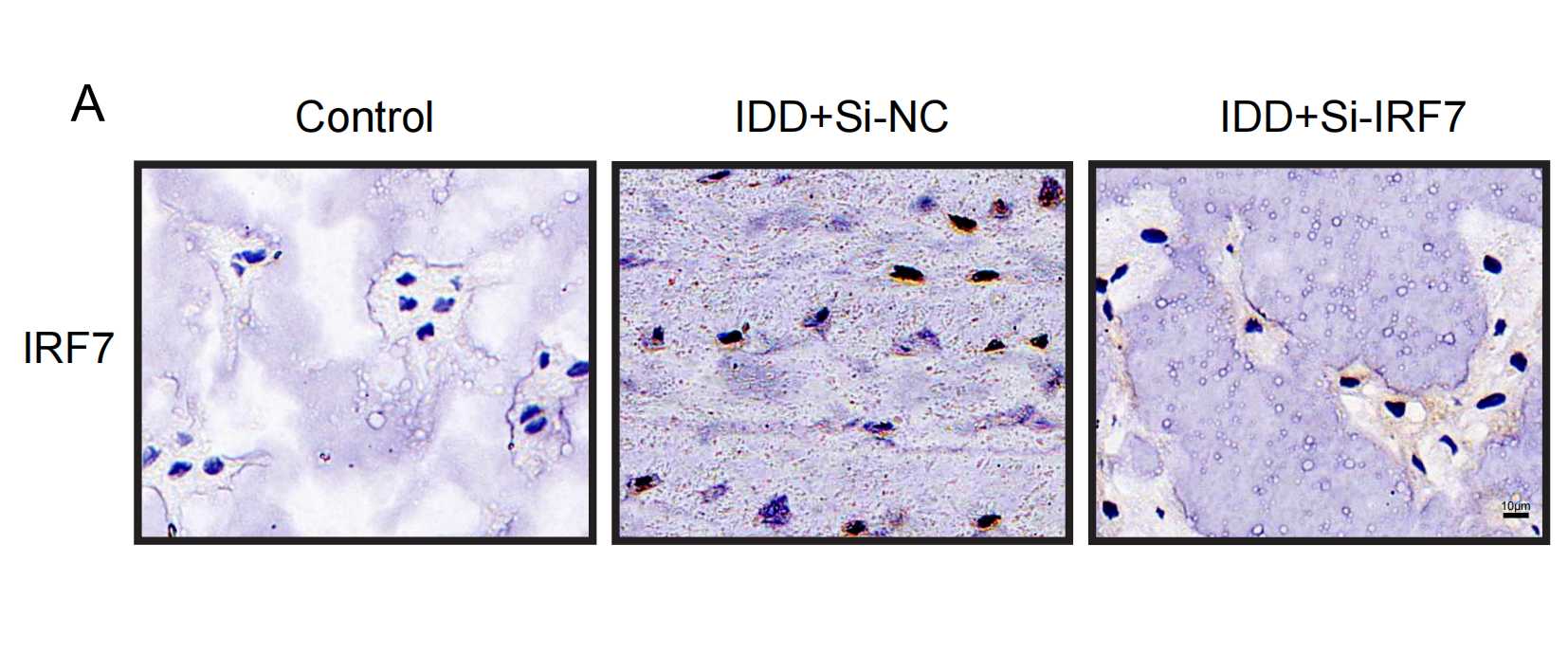

Supplement: Supplementary Figure 2 — IRF7 was successfully knocked down in vivo. [file Image2.jpeg]
